# Interferon-Alpha Decreases Cancer Stem Cell Properties and Modulates Exosomes in Malignant Melanoma

**DOI:** 10.3390/cancers15143666

**Published:** 2023-07-18

**Authors:** María Belén García-Ortega, Ernesto Aparicio, Carmen Griñán-Lisón, Gema Jiménez, Elena López-Ruiz, José Luis Palacios, Gloria Ruiz-Alcalá, Cristina Alba, Antonio Martínez, Houria Boulaiz, Macarena Perán, Michael Hackenberg, José Bragança, Sofia M. Calado, Juan A. Marchal, María Ángel García

**Affiliations:** 1 Biopathology and Regenerative Medicine Institute (IBIMER), Centre for Biomedical Research, University of Granada, 18016 Granada, Spain; mbcardiohuvn@gmail.com (M.B.G.-O.); eaparicioeaparicio@gmail.com (E.A.); glcarmex@gmail.com (C.G.-L.); gemajg@ugr.es (G.J.); jlpalaciosferrer@hotmail.com (J.L.P.); gloria_ra_1989@hotmail.com (G.R.-A.); hboulaiz@ugr.es (H.B.); mperan@ujaen.es (M.P.); hackenberg@go.ugr.es (M.H.); 2 Instituto de Investigación Biosanitaria de Granada (ibs. GRANADA), 18012 Granada, Spain; elruiz@ujaen.es; 3 Excellence Research Unit “Modelling Nature” (MNat), University of Granada, 18071 Granada, Spain; 4 Department of Oncology, Virgen de las Nieves University Hospital, 18014 Granada, Spain; cr.albatorres@gmail.com; 5 Department of Genetics, University of Granada, 18100 Granada, Spain; 6 Department of Biochemistry and Molecular Biology II, Faculty of Pharmacy, University of Granada, 18011 Granada, Spain; 7 GENYO-Centre for Genomics and Oncological Research-Pfizer/University of Granada/Andalusian Regional Government, 18016 Granada, Spain; 8 Department of Human Anatomy and Embryology, Faculty of Medicine, University of Granada, 18016 Granada, Spain; 9 Department of Health Sciences, University of Jaén, Campus de las Lagunillas SN, 23071 Jaén, Spain; 10 Department of Dermatology, Virgen de las Nieves University Hospital, 18014 Granada, Spain; antoniomartinezlopez@aol.com; 11 Algarve Biomedical Center Research Institute (ABC-RI), Universidade do Algarve, 8005-139 Faro, Portugal; jebraganca@ualg.pt (J.B.); sofiaamcalado@gmail.com (S.M.C.); 12 Faculdade de Medicina e Ciências Biomédicas, Universidade do Algarve, 8005-139 Faro, Portugal; 13 Champalimaud Research Program, Champalimaud Center for the Unknown, 1400-038 Lisbon, Portugal; 14 Department of Molecular Biology and Biochemistry III and Immunology, Faculty of Medicine, University of Granada, 18016 Granada, Spain

**Keywords:** interferon-α, malignant melanoma, cancer stem cells, exosomes, metabolomics, biomarkers

## Abstract

**Simple Summary:**

Malignant melanoma spreads to other organs and is resistant in part due to the presence of cancer stem cell subpopulations (CSCs). To combat these aggressive subpopulations, different therapies are being studied. Our study aimed to evaluate the anti-tumor effect of interferon-alpha (IFN-α) treatments on melanoma CSCs and explore potential biomarkers. We found that even low doses of IFN-α reduced CSC formation and stemness properties, and led to a significant decrease in the ability to form tumors in mice xenotransplants. IFN-α also modulated the expression of genes and microRNAs involved in several cancer processes and the metabolomics of released exosomes. Our data suggest further investigations of new dose and combination approaches with IFN-α in malignant melanoma.

**Abstract:**

Malignant melanoma (MM) can spread to other organs and is resistant in part due to the presence of cancer stem cell subpopulations (CSCs). While a controversial high dose of interferon-alpha (IFN-α) has been used to treat non-metastatic high-risk melanoma, it comes with undesirable side effects. In this study, we evaluated the effect of low and high doses of IFN-α on CSCs by analyzing ALDH activity, side population and specific surface markers in established and patient-derived primary cell lines. We also assessed the clonogenicity, migration and tumor initiation capacities of IFN-α treated CSCs. Additionally, we investigated genomic modulations related to stemness properties using microRNA sequencing and microarrays. The effect of IFN-α on CSCs-derived exosomes was also analyzed using NanoSight and liquid chromatography (LC-HRMS)-based metabolomic analysis, among others. Our results showed that even low doses of IFN-α reduced CSC formation and stemness properties, and led to a significant decrease in the ability to form tumors in mice xenotransplants. IFN-α also modulated the expression of genes and microRNAs involved in several cancer processes and metabolomics of released exosomes. Our work suggests the utility of low doses of interferon, combined with the analysis of metabolic biomarkers, as a potential clinical approach against the aggressiveness of CSCs in melanoma.

## 1. Introduction

Malignant melanoma (MM) is the most aggressive and life-threatening skin cancer with an increasing incidence worldwide. Over 90% of skin cancer deaths are due to MM, principally due to the high tendency to metastasize [1,2]. Many treatments have been tested to try to reduce MM early initiation of metastasis (chemotherapy, immunotherapy, radiotherapy, etc.) with modest or no efficacy. Several studies have shown that a postoperative adjuvant therapy with interferons (IFNs) might improve the recurrence-free survival (RFS) and overall survival (OS) of patients with high risk of suffering tumor recurrence [3]. In fact, systemic application of IFNs has been the only effective therapeutic option used in the dermato-oncological routine until the advent of immune-checkpoint inhibitors and BRAF/MEK-directed targeted therapy that are still under investigation in non-metastatic stages [4]. IFNs are a heterogeneous group of glycoproteins classified into type I (IFN-α, IFN-β), type II (IFN-γ) and type III. The IFN Receptor complex consists of two alpha chains (Type I receptor) in complex with Jak1 and Tyk2. These kinases phosphorylate Stat1 and Stat2, respectively. Finally, the Jak-STAT/IRFs pathway triggers the transcriptional modulation of more than 300 genes [5]. This activation leads to a wide range of biological responses, including antiviral, anti-proliferative and anti-tumoral effects. In addition, the stimulation of the cytotoxic activity of a variety of cells of the immune system by IFNs, increases the expression of tumor-associated surface antigens [6]. Despite the proven benefits of IFNs type I, questions regarding the optimal treatment regimen (dosage and duration), along with the significant side effects associated with this treatment, remain subjects of controversy and debate.

High risk MM is characterized by an extraordinary propensity for dissemination to distant organs and resistance to chemotherapy, in part due to the existence of MM cancer stem cells (CSCs) subpopulations. In fact, one of the major issues in the fight against cancer is that CSCs can survive treatments by slowly dividing, surpassing cytostatic drugs and probably, the immune system activity contributing to immune-checkpoint inhibitors and BRAF/MEK-directed targeted therapy resistances [7,8]. CSCs represent a small fraction of the total cell population in a solid tumor and are defined by their ability of self-renewal and to produce tumor heterogeneity [9]. Expression of tissue type-specific cell surface markers has been used to isolate and enrich CSCs in vitro from different tumors. MM CSCs overexpress CD20, CD44, CD133 and the membrane transporter ABCB5, have high activity of the enzyme aldehyde dehydrogenase (ALDH) and have been shown, in animal models, to induce primary tumor initiation [10]. The high metastatic potential of MM is facilitated in part by the interaction between MM-CSCs and the tumor microenvironment (TME). In fact, CSCs secrete soluble factors and extracellular vesicles (EVs), which include exosomes, enclosing specific lipids, proteins and RNAs that contribute to the pre-metastatic niche formation and, consequently, tumor spread [10,11]. Thus, targeting CSCs subpopulation within MM would reduce the fatal metastatic potential of the skin cancer cells.

Here, we have analyzed the effect of low and high doses of IFN-α-treatment on melanospheres enriched in CSCs subpopulations from the A375 MM established cell line and the MEL-1 primary cell line. To do so, we firstly measured by flow cytometry the ALDH activity, the side population and the specific stemness surface markers expression. Secondly, we evaluated the clonogenic ability of treated cells by soft-agar assay, the migration capacity of cells by wound-healing assay and the anti-CSCs properties in mice xenotransplant models. Moreover, we studied by miRNAseq and microarrays the effect of IFN-α on the expression of miRNAs related to stemness and the modulation of the expression of tumor related genes. Finally, we studied the effect of IFN-α on exosomes released from MM CSCs. To do so, we characterized and compared the protein content of MM CSCs released exosomes, of control CSCs versus IFN-α treated CSCs, by NanoSight, TEM, SEM Western blot and LC-HRMS-based metabolomic analysis.

Our work suggests the utility of low dose of Interferon together with the analysis of metabolic biomarkers as a potential clinical approach against the aggressiveness of CSCs in melanoma. This novel approach, together with current therapies, could improve MM treatment.

## 2. Materials and Methods

### 2.1. Cell Culture, CSC Enrichment and IFN-α Treatments

MM cancer cell lines were obtained from A375 and MEL-1 cell cultures. The line A375 was obtained from American Type Culture Collection (ATCC). Human primary MEL-1 cell line derives from a malignant metastatic melanoma (stage M1a) skin biopsy (BBSPA-Mel#1), and was provided by the Biobank of the Andalusian Public Health System (Spain). This cell line is hipotriploid (complex karyotype with multiple numerical and structural chromosome abnormalities), MelA positive, p53 positive and S100 positive, and with high tumourigenic ability. Melanoma adherent cells lines were maintained in standard culture conditions in a humid incubator at 37 °C and 5% CO_2_, with DMEM (Dubelcco’s Modified Eagle’s medium, Sigma-Aldrich, St. Louis, MO, USA) supplemented with 10% heat inactivated fetal bovine serum (FBS) (Gibco, Grand Island, NY, USA) and 1% Penicillin/Streptomycin (P/S) (Sigma-Aldrich) in 75 cm^2^ flask culture (Nunc, Roskilde, Denmark) unless otherwise indicated. FBS was inactivated by heating at 56 °C for 45 min. Cells were assayed for mycoplasma contamination. 

Enriched MM CSCs subpopulation were obtained after cultivation as primary and secondary spheroids in serum free medium and under anchorage-independent conditions as described by Jiménez et al., 2018 [10]. Briefly, for primary spheroids culture, cells were plated in serum-free spheres culture medium (DMEM:F12, 1% P/S, B27, 10 µg/mL ITS, 1 µg/mL Hydrocortisone, 4 ng/mL Heparin, 20 ng/mL EGF, 10 ng/mL FGF, 10 ng/mL IL6, 10 ng/mL HGF) in ultra-low adherence 6-well plates (Corning, Corning, NY, USA). For the secondary spheres culture, cells from primary spheroids were collected by centrifugation (1500 rpm for 10min), and, then, the pellet was resuspended in DMEM:F12 sphere medium and mechanically disrupted with a pipette and by syringing three to five times through a sterile 25-gauge needle. After that, cells were plated and incubated for 72 h in spheres culture medium in ultra-low adherence 6-well plates and treated with different IFN-α concentrations: 2000 IU/mL (low dose) and 20,000 IU/mL (high dose). The IFN-α was facilitated by Hospital Pharmacy Service at Hospital Universitario Virgen de las Nieves (Granada, Spain) under the commercial name of INTRON A^®^ (Interferon ALFA-2b for Injection −10 million I.U. per 1 mL × 1 vial- of MSD laboratories) [12].

### 2.2. Sphere-Forming Assay

To determine the self-renewal ability of the MM CSCs population, sphere-forming assay was performed [13]. For the secondary sphere-forming assay, 2.5 × 10^5^ single cells derived from primary spheroids were plated and resuspended in spheres culture medium in ultra-low adherence 6-well plates (Corning) and treated with the same IFN-α concentration detailed above. Secondary spheres > 75 µm diameter were counted after 3 days by light microscopy. Diameters were measured using the ImageJ software. 

### 2.3. Colony-Formation Assay

The clonogenic capability of MM CSCs were determined by colony-formation assay in soft agar as previously described [13] with minor modifications and treated with IFN-α. Briefly, 10^4^ cells coming from secondary spheroids were seeded in 0.4% cell agar base layer, which was on top of 0.8% base agar layer in 6-well culture plates. Then, cells were incubated for a further 27 days at 37 °C and 5% CO_2_, adding 100 µL of DMEM (10%FBS, 1% P/S) every 1–2 days. Cell colony formation was then examined under a light microscope after staining with 0.1% Iodonitrotetrazolium Chloride (Sigma-Aldrich) [13]. The size of colonies were measure using ImageJ™ software.

### 2.4. Aldefluor Assay and Phenotypic Characterization by Flow Cytometry

The analysis of CD20, CD44 and CD133 surface markers and the ALDH1 activity were performed using a Becton Dickinson FACSCanto II flow cytometer from the CIC Scientific Instrumental Centre (University of Granada) as previously described [13]. Briefly, aldefluor assays (Stem Cell Technologies, Vancouver, BC, Canada) to detect ALDH1 activity in viable cells were performed according to manufacturer’s instructions. Cell lines were suspended in aldelfuor assay buffer containing ALDH1 substrate (BAAA, 1 μmol/L per 1 × 10^6^ cells) and incubated during 45 min at 37 °C in darkness. Dethylaminobenzaldehyde (DEAB) was used as an ALDH1 inhibitor to set ALDH1 gates. The brightly fluorescent ALDH1-expressing cells were detected in the green fluorescent channel (520–540 nm). 

Cell surface levels of CD20, CD44 and CD133 were determined with anti-human antibodies CD20-allopycocyanin (APC), CD44-phycoerithrin (PE) and CD133-allopycocyanin (APC) (MiltenyiBiotec, BergischGladbach, Germany), respectively. All samples were analyzed on a FACS CANTO II (BD Biosciences, San Jose, CA, USA) using the FACS DIVA software. 

### 2.5. Side Population Assays

Hoechst 33342 exclusion (Side Population) assays were carried out as previously described to analyze cells overexpressing ABC transporters. Melanospheres were stained with Hoechst 33342 (Sigma-Aldrich) dye. As negative controls, Verapamil (Sigma-Aldrich) was used for maintaining the efflux channel closed inhibiting the capacity to efflux Hoechst 33342 by cells [14]. The brightly fluorescent cells were measured by flow cytometry in Hoechst blue (440/40) and Hoechst red (695/40) of a FACScan Aria III (BD Biosciences) using FACS DIVA software from the CIC Scientific Instrumental Centre (University of Granada). Cells with the ability to efflux Hoechst 33342 were considered as the side population (SP) [14].

### 2.6. Wound-Healing Assay

A375 and MEL-1 MM cells lines were seeded in 6-well low attachment plates and IFN-α treatment was added at 2000 and 20,000 IU/mL. Wounds were created by scraping monolayer cells (after disrupted by syringing and plated in 6-well) with 200 µL pipette tip, and non-adherent cells were washed off with medium. At 0, 24, 48 and 72 h after the creation of wounds, IFN-treated and control non-treated cells were observed and photographed with a 10× objective by light microscopy. Wound distances were measured at each time point and expressed as pixels-area^2^ migration of wound closure by comparing the zero time. Image-J software was used to quantify the wound area [15]. Cell cycle and apoptosis analysis are explained in Supplementary Materials.

### 2.7. Microarray Profiling and Analysis

A375 and MEL-1 MM CSCs were treated with IFN-α for 24 h 2000 IU/mL, which will be referred to as low dose, hereafter. Non-treated cells were used as control. Total RNA was extracted using Qiagen extraction kit, according to the manufacturer’s protocol. Transcriptome microarray profiling was carried out using Clariom™ S Assay, Affymetrix Human arrays according to the Affymetrix standard protocol. Data analysis was performed using TAC 4.0 (Transcriptome Analysis Console) Thermofisher software, R and the CRAN package VennDiagram [16]. In order to establish relationships between selected genes, String data-base was used [17]. 

### 2.8. miRNA NGS Profiling of MM CSCs and Differential Expression Analysis

For each cell line, A375 and MEL-1, three different types of libraries were prepared: (i) untreated MM adhered (non-stem-like) cells; (ii) untreated MM CSCs; and (iii) IFN-α stimulated MM CSCs. Treated MM CSCs were exposed to a low dose of IFN-α for 24 h prior to RNA extraction. Each of the cell-line-condition combinations described were performed and profiled in duplicate (6 conditions, 12 RNA libraries). For sequencing library preparation, 1 μg total RNA was used and libraries were prepared according to the TruSeq Small RNA Sample Prep Kit (Illumina) protocol with automated pooled library size selection using Pippin Prep (Thermo Fisher Scientific). Concentration and size profile of the sequencing libraries were measured using Bioanalyzer (DNA 1000 assay) and KAPA library quantitation kit qPCR determined that the pool concentration was 11.91 nM. The pool of samples was run in one lane on a HiSeq2500 instrument (Illumina) for 50 cycles. Resulting sequencing files were processed with sRNAbench [18] and using miRBase (release 22) as miRNA annotation [19]. Quality control was performed using mirnaQC with all samples passing minimum quality criteria and no outliers detected [20].

Count values were normalized using the Variance Stabilizing Transformation and differential expression analysis was performed using DESeq2 [21]. miRNAs were considered to be differentially expressed for fold changes above 2 or below 0.5 and False Discovery Rate (FDR) corrected *p*-values below 0.05. Consistently under- or overexpressed genes across cell types were systematically detected for each given comparison and included in downstream validation. Quantitative real time-PCR (qRT-PCR) is explained in Supplementary Materials.

### 2.9. In Vivo Tumor Xenograft Assays

CSCs from A375 and MEL-1 MM cell lines were used for xenograft assays. After 72 h of treatment with low dose of IFN-α, 500 cells were injected in 0.05 mL matrigel and 0.05 mL of culture medium by subcutaneous injections to 8-week-old NOD scid mice gamma (NOD.Cg-Prkdcscid Il2rgtm1Wjl/SzJ, NSG). Tumor growth was assessed twice a week using a digital caliper and the tumor volume was calculated by the formula V = length^2^ × width × π/6. Animal experimentation was performed according to the protocols reviewed and approved by the Institutional Animal Care and Use Committee of the University of Granada (13/08/2020/095). After 375 and 91 days, in A375 and MEL-1, respectively, animals were sacrificed and the tumors were sectioned and embedded in paraformaldehyde (PFA).

### 2.10. Immunofluorescence Assay

Tumors of different conditions were immersed in 4% PFA in 0.1 M PBS for 4 h at 4 °C, washed in 0.1M PBS and embedded in paraffin in an automatic tissue processor (TP1020, Leica, Germany). The paraffin blocks were cut into 4 mm sections and subjected to immunofluorescence assay. Antigen retrieval was performed at 121 °C for 15 min in a sodium citrate buffer solution (pH 6.0), and then, sections were deparaffinized with xylene and hydrated with decreasing alcohol concentrations (absolute to 70%). 

The tissue sections were then incubated with rabbit anti-p75 antibody (Abcam), rabbit anti-SAMD9 antibody (Abcam), rabbit anti-CD133 antibody (Abcam) and mouse anti-CD44 (Santa Cruz Biotechnology) in phosphate-buffered saline (PBS) containing 1% bovine serum albumin (BSA) overnight at 4 °C. Next day, samples were washed thrice with PBS and incubated with the secondary antibodies (Alexa) for 1 h at RT, after washing thrice with PBS and mounted with DAPI-containing mounting medium. Negative control tissue sections were prepared by omitting the primary antibody. Observation under light microscopy and digital image acquisition were carried out with an inverted microscope (Nikon H550s). Its immunofluorescence intensity was qualified using ImageJ™ software (NIH Image, Bethesda, MD, USA).

### 2.11. Exosome Isolation and Purification

Exosomes were collected from culture supernatants secondary spheres generated from A375 and MEL-1 MM CSCs and from serum of MM patients by ultracentrifugation as previously described by Costa-Silva et al. with minor modifications [22]. We set out from ~200 mL of supernatant fractions collected from cell cultures at 72 h with incubation of low dose IFN-α in each purification procedure. Supernatants were first centrifuged at 500× *g* for 10 min. Next, the pellet was discarded and the remaining supernatant was ultracentrifuged at 12,000× *g* for 20 min in a JS-24-38 rotor (Beckman Coulter Inc., Fullerton, CA, USA). The pellet contained microvesicles (MVs), which were resuspended in 100 µL of Dulbecco’s Phosphate Buffered Saline modified without calcium chloride and magnesium chloride and sterile filtered (modified PBS; Sigma-Aldrich, St. Louis, MO, USA), whereas the supernatant was ultracentrifuged at 100,000× *g* for 70 min. The resulting pellet was washed in 35 mL PBS and pelleted again by ultracentrifugation at 100,000× *g* for 70 min. Finally, exosomes were obtained in the pellet, which was resuspended into 100 µL of modified PBS and stored frozen at −80 °C for further analyses. Repeated freezing and thawing of the exosome suspensions were avoided. Transmission and Scanning Electron Microscopy Atomic Force Microscopy are explained in Supplementary Materials.

### 2.12. Exosome Size Analysis

Size analyses were performed on NanoSightNS500 instruments (Malvern Instruments, UK). The instrument was equipped with a 488 nm laser, a high sensitivity CMOS camera and a syringe pump. Exosomes were diluted 1:1000 in PBS buffer to obtain a concentration range (1–10 × 10^8^ particles/mL). The measurements were analyzed using the NTA2.3 software (Malvern) after capturing 3 videos of 60 s. Inmunogold Labeling by Transmission Electron Microscopy are explained in Supplementary Materials.

### 2.13. Western Blot Analysis

The final pellets of cell culture supernatants of CSCs were resuspended in 100 μL of PBS and stored at 4 °C for further protein quantification. The protein concentrations were measured using the BCA Protein Assay Kit (Pierce, Rockford, IL, USA) according to manufacturer’s instructions. Proteins extracts (30 μg) were denatured at 95 °C for 5 min in loading buffer (containing Tris—pH 6.8, SDS, glycerol, β-mercaptoethanol and bromophenol blue). Proteins were subjected to 4–20% Mini-PROTEAN TGX (Bio-Rad, Hercules, CA, USA) gel together with Precision Plus ProteinTM Kaleidoscope Prestained Protein Standards (Bio-Rad, USA). The samples were transferred to a nitrocellulose membrane (Trans-Blot, Mini Format, Bio-Rad) using a transfer apparatus according to the manufacturer’s protocols (standard program: 25 V for 30 min) (Bio-Rad). 

After incubation with 5% skimmed milk in PBS-Tween 0.1% for 1 h at room temperature, the membranes were incubated overnight with antibodies against CD9 (dilution 1/1500, eBioscience), CD63 (dilution 1/500, Santa Cruz Biotechnology), p75 (dilution 1/500, Abcam), Hsp-70 and Alix (dilution 1/1000, Cell Signaling). Membranes were then incubated with conjugated goat anti-mouse secondary antibody and goat anti-rabbit secondary antibody for 2 h and signals were detected using the ECL-PLUS (Amersham Biosciences). The bands were visualized with medicals photographic films (AGFA) or detected using the Infrared Odyssey Imager (LI-COR Biotechnology, Lincoln, NE, USA). 

### 2.14. LC-HRMS Analysis of Exosomes

The metabolomic analyses of exosomes isolated from cell culture supernatant were performed in Fundación MEDINA (Centro de Excelencia en Investigación de Medicamentos Innovadores en Andalucía) as described by García-Fontana et al., with minor modifications [23]. Sample preparation for LC-HRMS analysis was performed as follows: Exosome samples were thawed on ice, vortexed and kept at 4 °C during the analytical process. Proteins were withdrawn using methanol (1:3), shaken, sonicated (1 min) and shaken again. Samples were then centrifuged at 13,300 rpm for 15 min at 4 °C. Supernatants were collected and dried under an N2 air stream. Dried samples were reconstituted in 90 μL of mobile phase (50% H_2_O and 50% acetonitrile at 0.1% of formic acid) and transferred to the analytical vials. Then, samples were analyzed in triplicate using AB SCIEX TripleTOF 5600 quadrupole-time-of-flight mass spectrometer (Q-TOF-MS) (AB SCIEX, Concord, Canada) coupled to a high-performance liquid chromatography (HPLC) system, in positive electrospray ionization (ESI) mode. 

Previous to HRMS analysis, chromatographic separation was carried out by an Agilent Series 1290 LC system (Agilent Technologies), equipped with a reverse phase Atlantis T3 HPLC C18 column (C18: 2.1 mm × 150 mm, 3 mm) (Waters). Samples were injected randomly (5 μL per sample) into the HPLC system. Blank solvent (BS) and quality control (QC) samples were also injected throughout the sequence run. The QC samples were prepared by pooling an equal volume of all exosome samples and injected every five samples in order to assess the stability and performance of the system. The BS samples were also run interspersed in the sequence to detect possible impurities of the solvents or extraction procedure and to check carryover contamination from intense analytes. Generic parameter settings for chromatographic separation and MS detection were used to obtain specific metabolomic fingerprints of the exosome preparations. HRMS analysis was performed using an information-dependent acquisition (IDA) method to collect full scan MS and MS/MS information simultaneously. The method consisted of high-resolution survey spectra from *m*/*z* 50 to *m*/*z* 1600 and the 8 most intense ions were selected for acquiring MS/MS fragmentation spectra after each scan. An Automated Calibration Delivery System performed an exact mass calibration prior to each analysis. 

Data set creation: PeakView software (AB SCIEX) was used in order to evaluate the analytical drift in terms of mass and retention time shift. MarkerView software (version 1.2.1.1, AB SCIEX) was used for processing the LC-HRMS raw data. This software performs peak detection, alignment and data filtering, generating a feature table which defines measured *m*/*z*, retention time (RT) and integrated ion intensity. An automated algorithm in the RT range 0.8–19 min and *m*/*z* range 50–1600 was used for data mining. The intensity threshold of extraction was established at 50 counts per second. RT and *m*/*z* tolerances of 0.1 min and 15 ppm, respectively, were used for peak alignment. Background noise was removed by using a specific tool of MarkerView software. The analytical replicates of each sample were averaged. 

Analytical validation: QC distribution on PCA plot was used for analytical validation prior to the following analysis. Variables with unacceptable reproducibility (RSD > 30%) or detected in less than 50% of QC samples were also rejected from the data matrix.

Data treatment: Statistical analyses were carried out using MetaboAnalyst 4.0 Web Server [24] as previously described [23]. Briefly, after dataset creation, raw data were normalized (median normalization), transformed (cube root transformation) and scaled (Pareto scaling) in order to achieve a more Gaussian type distribution [25]. Then, filtering according to significant differences was performed based on statistical analysis including both univariate (UVA) and multivariate (MVA) in order to identify variables (metabolites) that were significantly different between the groups compared. For UVA, a first double filtering procedure with *t*-test (*p*-value < 0.05) and fold-change (FC > 1.5) was applied to identify differentially expressed mass signals between BS and exosome samples and therefore discard them as background noise, preserving the peaks from true biological samples.

Then, a *t*-test based filtering (*p*-value < 0.05) was used to detect differential metabolites between the sample groups, providing a quality criterion to evaluate variable relevance for further data analysis. For MVA, principal component analysis (PCA) and partial least squares regression (PLS-DA) were carried out after *t*-test filtering. PCA was applied to assess quality of the analytical system performance. PLS-DA allowed discriminating variables that are responsible for variation between the comparison groups. For statistical validation, quality description by goodness of fit (R2) and goodness of prediction (Q2) was used. A powerful model for diagnostics should show high values of R2 and Q2 but also not vary more than 0.2–0.3. For metabolomics data, R2 > 0.7 and Q2 > 0.4 are considered acceptable values [25]. The models were also validated using 10-fold cross validation.

### 2.15. Statistical Analysis

All data are presented as the mean ± standard deviation. Differences between groups were analyzed for statistical significance using the two-tailed Student’s *t*-test. *p*-value of 0.05 was accepted as the statistical significance level. The different statistical studies have already been reflected in each section of materials and methods.

## 3. Results

### 3.1. IFN-α Reduces Melanospheres Proliferation and Colony Formation Capacity of MM CSCs

**Figure 1:**
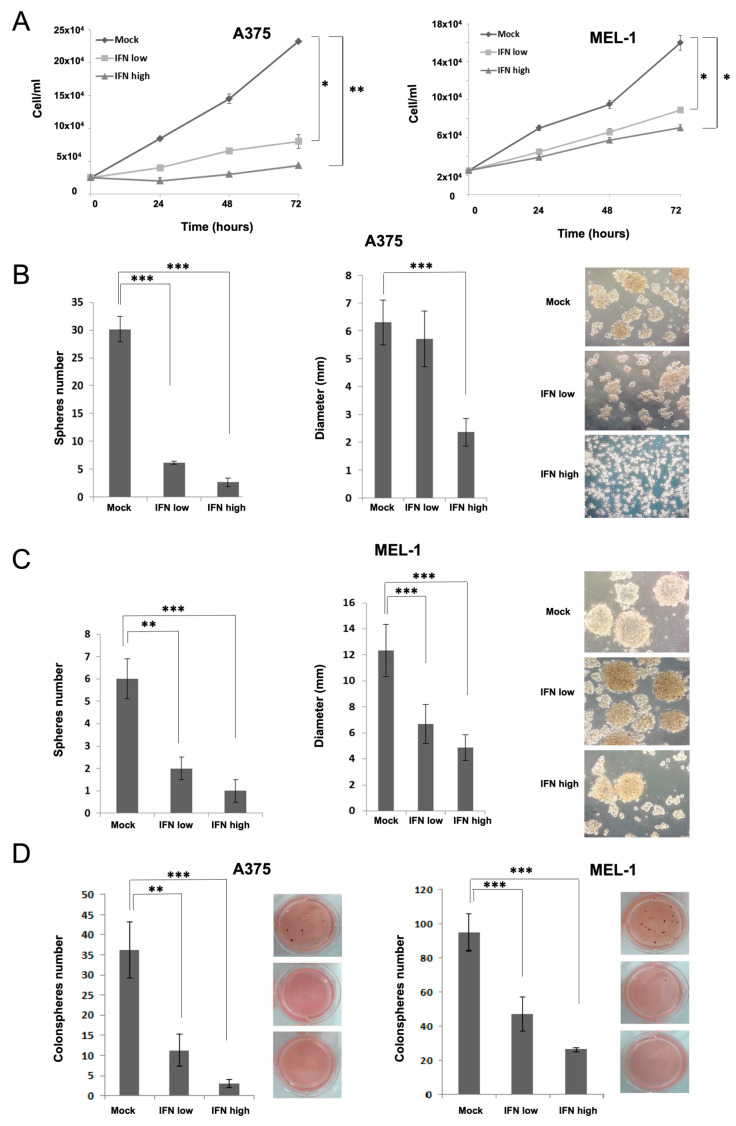
Proliferation assay, tumorsphere and colony-forming ability of MM CSCs enriched subpopulations. (**A**) Proliferation curves of A375 and MEL-1 CSCs subpopulations after treatment with low and high doses of IFN-α compared with controls non-treated cells (Mock). The initial cell number plated was 25,000 cells per well in all conditions; (**B**,**C**) Number of secondary spheres formed by A375 and MEL-1 cells after treatment with low and high doses of IFN-α compared with Mock cells. Melanospheres were counted after 3 days under light microscopy; the diameter of spheres from A375 cell line and MEL-1 cell line were measured by ImageJ software. Representative light microscopy (4×) images of spheres formed have been included. (**D**) Representative optical image of the colonies formed by A375 and MEL-1 cells from secondary spheroids (previously IFN or Mock-treated), after 37 days of soft agar culture in P6 well plates, stained with 0.1% Iodonitrotetrazolium Chloride. Data are graphed as mean ± SD from experiments carried-out by triplicates (*** *p* < 0.001; ** *p* < 0.01; * *p* < 0.05).

Following CSCs enrichment by secondary spheres growth of established A375 MM cells and Mel1 primary tumor cells, we analyzed the effect of IFN-α on CSCs characteristics, such as sphere forming ability, proliferation rate and clonogenic capacity by colony-formation assay in soft agar (Figure 1). CSCs from A375 and MEL-1 cell lines treated with low and high doses of IFN-α showed lower proliferation rates respect to mock treatment (Figure 1A). In addition, we observed a significant decrease in the number and diameter of spheres as generated from A375 and MEL-1 CSCs after IFN-α treatments (Figure 1B,C). Accordingly, with these results, non-treated melanospheres showed a high capacity to form colonies in soft agar assay, which were significantly reduced after IFN-α treatments for both cell lines (Figure 1D).

### 3.2. IFN-α Reduces Stemness Properties

**Figure 2:**
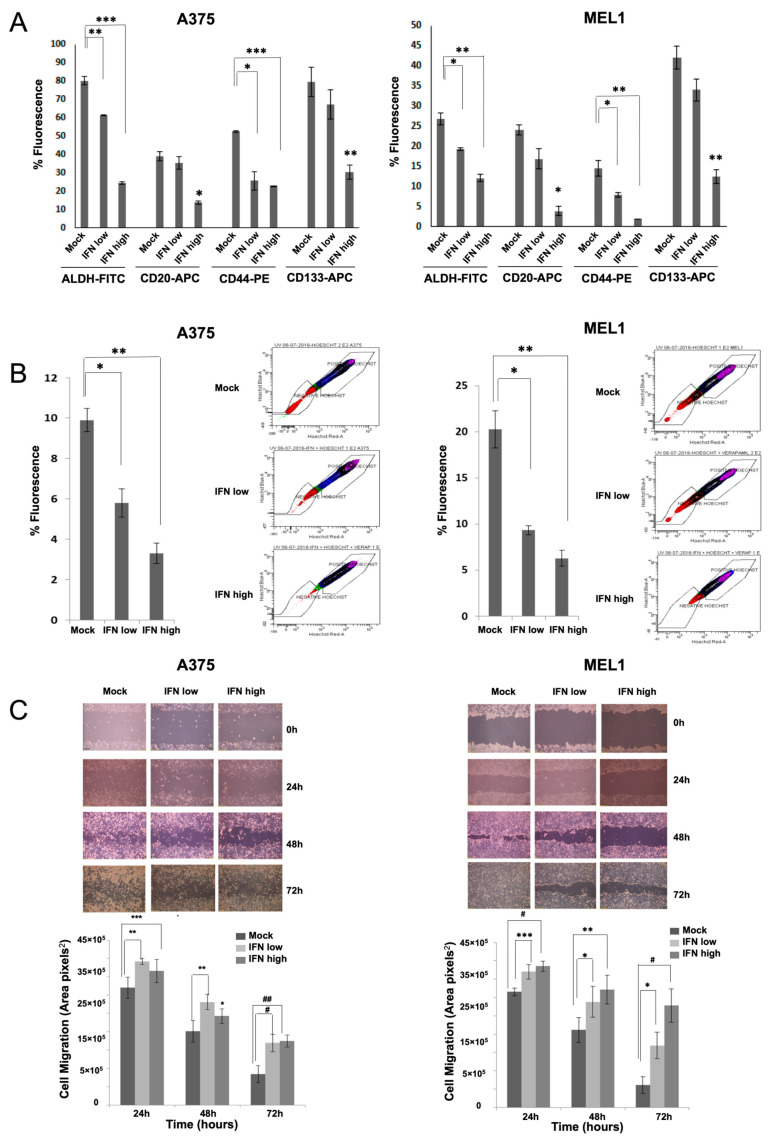
Effect of IFN-α treatment on the stemness properties of MM CSCs enriched subpopulations. (**A**) Comparative analysis for ALDH1 activity, CD20+, CD44+ and CD133+ expression in A375 and MEL-1 CSCs subpopulations analyzed by flow cytometry after treatment with low and high dose of IFN-α. The data were analyzed by *t*-test, *** *p* < 0.001; ** *p* < 0.01; * *p* < 0.05. (**B**) Effect of IFN-α on SP percentage in A375 and MEL-1 CSCs subpopulations; (**C**) Wound healing assay on A375 and MEL-1 CSCs subpopulations. Cells migration was quantified by measuring the wound closure area in pixels at 24, 48 and 72 h with ImageJ software and graphed. Representative optical images (10×) show the cells migration. The data were analyzed by *t*-test, ## *p* < 0.0001; # *p* < 0.0005; *** *p* < 0.001; ** *p* < 0.01; * *p* < 0.05.

To evaluate the effect of IFN treatments on the MM CSCs properties, A375 and MEL-1 secondary spheres were characterized by measurement of specific markers expression such as CD20, CD44 and CD133, and ALDH1 activity, in presence and absence of IFN-α (Figure 2). ALDH1 activity and stemness surface markers expression decreased after IFN-α treatments in both CSCs subpopulations. However, the differences were only significant with high doses of IFN treatment (Figure 2A). In addition, side population (SP) fraction was analyzed using the Hoechst 33342 staining protocol in melanospheres, in absence or presence of IFN-α treatments, and revealed a significant reduction for both subpopulations after treatments with low and high doses (Figure 2B).

Since CSCs have higher ability to invade and migrate than other cancer cells [10], we evaluated the effect of IFN-α treatments on cell migration performing a wound healing assay (Figure 2C). Both CSCs subpopulations migrated faster and closed the gap made by the scratch in absence of IFN-α treatment in comparison to treated cells, indicating an impairment of cell migration upon IFN-α treatments. Moreover, the difference between low and high doses of IFN-α treatments was less marked in A375 CSCs than in MEL-1 CSCs.

Cell cycle analyses of melanospheres cultured with both doses of IFN-α showed an accumulation of cells predominantly in S phase, with a concomitant restriction of cells on G1 and G2 phases for A375 and in G1 for MEL1-1 CSCs subpopulations (Supplementary Figure S1A). Despite the apoptotic-resistance offered by the CSC-enriched subpopulations, we were able to detect a significant increase in the levels of total apoptosis in our models after IFN-α treatments (Supplementary Figure S1B). 

### 3.3. Gene Expression Profile Changes and Effects on Selected miRNAs after MM CSCs IFN-α Treatment

**Figure 3:**
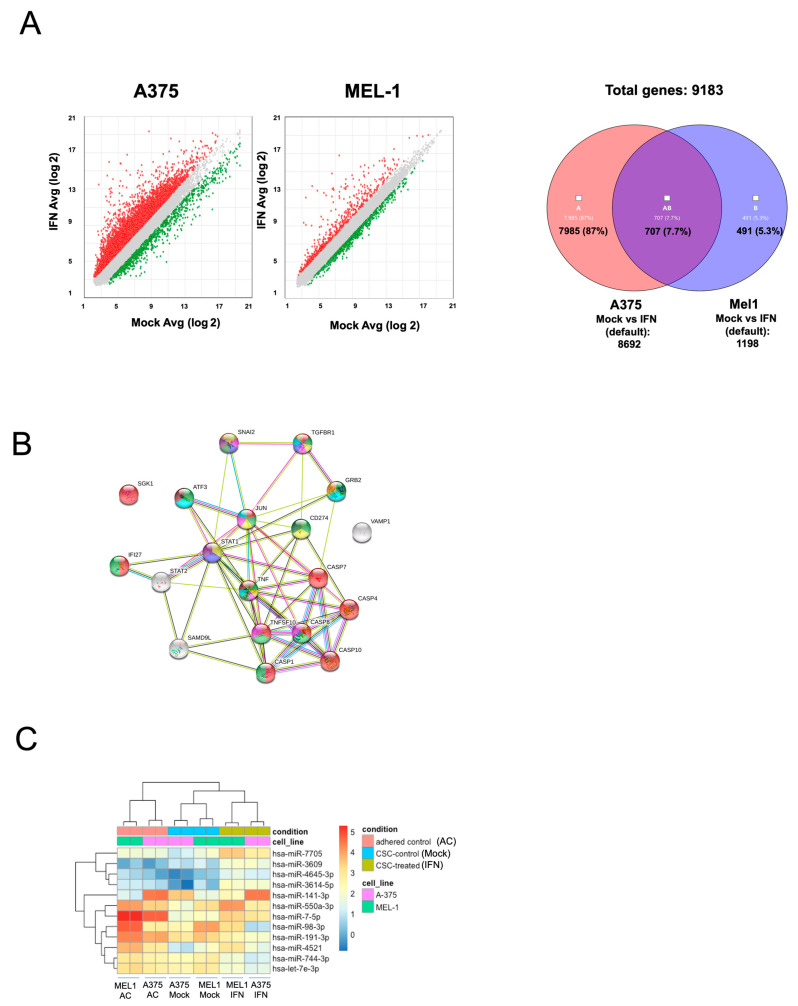
Array and RNAseq analysis of MM CSCs. (**A**) Gene expression scatter plots of control versus 24 h IFN-α treated CSCs in A375 and MEL-1 cell lines. Up- and downregulated genes are shown in red and green, respectively. Number of common up- and downregulated genes (Fold Change > 2 and FDR < 0.05) between A375 and MEL-1 secondary spheres after 24 h of IFN-α treatment. (**B**) Functional relationships among selected genes as displayed by String database. (**C**) Heatmap of expression values (log2 Read Per Million) of selected miRNAs in A-375 and MEL-1 secondary spheres after 24 h of IFN-α treatment.

Gene expression profiling and miRNA seq analysis for CSCs was used to understand the molecular changes underlying low dose IFN-α stimulation and to explore potential biomarkers. To this end, we used first a cDNA microarray platform with probes for 21,448 different genes. Differentially expressed genes were identified (cut-off values > 2- or <−2-fold change and *p* < 0.05) (Figure 3). For CSCs from A375, 8692 genes passed filter criteria; among these genes, there were 1099 up-regulated and 1409 down-regulated after IFN-treatment. For CSCs from MEL-1, 1198 genes passed filter criteria; among these genes, there were 252 up-regulated and 403 down-regulated after IFN-treatment (Figure 3A and Figure S2). Genes related to cancer processes, such as apoptosis (CASP1, CASP4, CASP7, CASP8, CASP10, SGK1 and TNFSF10), proliferation (STAT1, CD274, TNF, JUN), migration (GRB2), vesicle related genes (VAMP), MAPK (ATF3, JUN, GRB2) or Notch signaling pathways (STAT1) were shortlisted from the list of differentially expressed genes and used to establish a functional relationship using the String resource (Figure 3B) [26]. The final list was made up of 17 genes (Supplementary Tables S1 and S2), being the gene SAMD9L the one that showed striking expression in both CSCs subpopulations. Proteins involved in CSCs-related pathways such as SAMD9L, CD133, p75-NGFR and CD44 presented the same trend of expression in both cell lines after IFN-α treatments (Supplementary Figure S2C).

The miRNA seq analysis considered three different types of libraries for each cell line as shown in the Material and Methods section. We selected miRNAs considered to be differentially expressed for fold changes above 2 and those involved in different tumor processes and stemness properties (Figure 3C). In CSCs generated from A375 and MEL-1, miR-7-5p, miR-141-3p, miR-425, miR-550a, miR-3614-5p, miR-4521 and miR-4645 were overexpressed after IFN-treatment in comparison to Mock conditions. In contrast, we observed a lower expression of miR-98-5p, miR-191, miR-744-3p and let-7e-3p in both CSCs after IFN-treatment (Figure 3C). The regulation of the expression of these miRNAs following IFN-α treatment in both lines was validated by qPCR (Supplementary Figure S2D). 

### 3.4. IFN-α Reduces the Tumorigenicity of MM CSCs in Xenograft Mice

**Figure 4:**
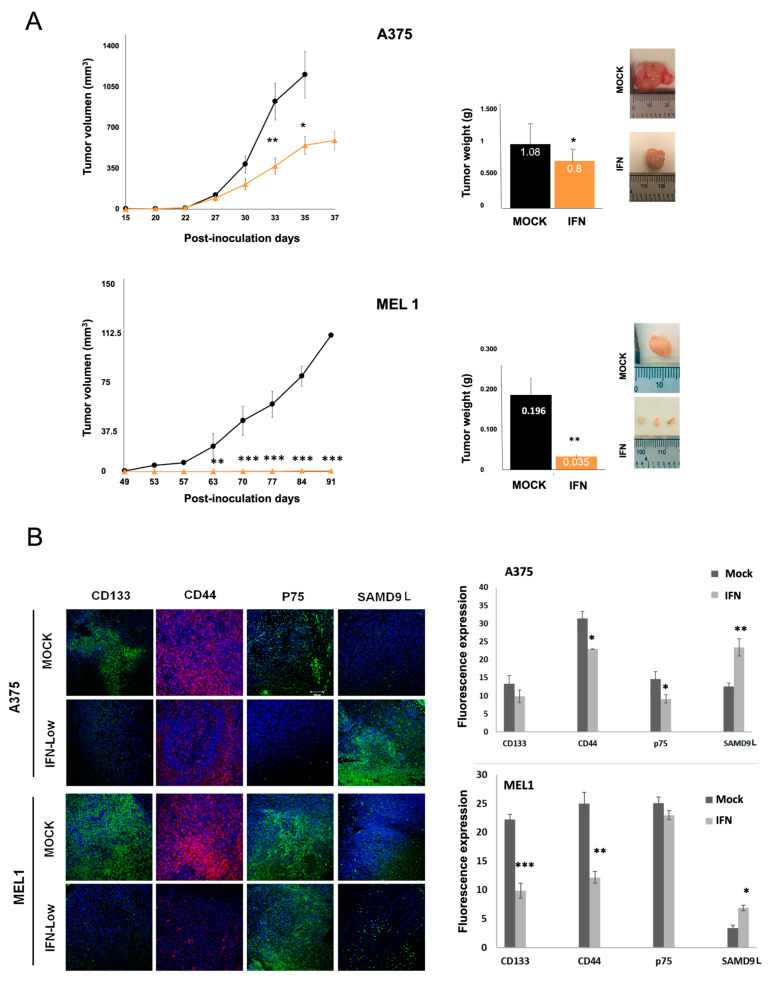
In vivo tumor formation by IFN-α pre-treated MM CSCs. (**A**) Tumor volume and weight of MM tumors formed in NSG mice after inoculation of A375 CSCs subpopulations and MEL-1 CSCs subpopulations. Representative images of tumors are shown. Data are shown as mean ± SEM statistical analysis Student’s test comparison IFN vs. Mock. (**B**) Histopathology of tumors formed by IFN-α pre-treated spheres. Representative immunofluoresence images for CD133, CD44, p75 and SAMD9L of tumors. Original magnification: 20×. Scale bar = 100 µm; Graph of the quantification of the fluorescence intensities. The average fluorescence intensities were calculated from three parallel immunofluorescence images. Statistical significance indicated * (*p* < 0.05), ** (*p* < 0.01), *** (*p* < 0.001).

To test in vivo the ability of IFN-α to inhibit the initiating tumor capacity of MM CSCs, secondary melanospheres of A375 and MEL-1 were treated with low dose IFN-α during 72 h and after that, 500 viable cells/mL were injected into both subcutaneous flanks of 20 NSG mice for every cell line. The effect of the treatment on tumor volume and weight is graphed in Figure 4.

Tumors generated by CSCs from A375 emerged 27 days after the injection in control and IFN-α pre-treated spheres; however, tumor from control CSCs displayed significantly higher volume and weight than tumors induced from IFN-treated CSCs (Figure 4A, upper panel). Tumors generated by CSCs from MEL-1 emerged 53 days after the injection, although surprisingly, they were barely detected in mice injected with IFN-α treated CSCs showing a significant reduction in mean tumor weight (Figure 4A, bottom panel). 

Finally, immunostaining for classical stemness markers (CD44, CD133 and p75) and the identified SAMD9L protein was performed in excised tumors’ sections showing a different level of fluorescence in tumors from IFN-α pre-treated spheres (Figure 4B). The signal reduction was significant for CD44 and p75 in A375-derived tumors and for CD133 and CD44 in MEL-1-derived tumors. In contrast, a significant increase in SAMD9L expression was observed in tumors induced from both A375 and MEL-1 cells treated by IFN-α, which is in agreement with the previous genomic results (Figure 4B).

### 3.5. IFN-α Interferes with EVs Secretion via Exosomes in MM CSCs Subpopulations

**Figure 5:**
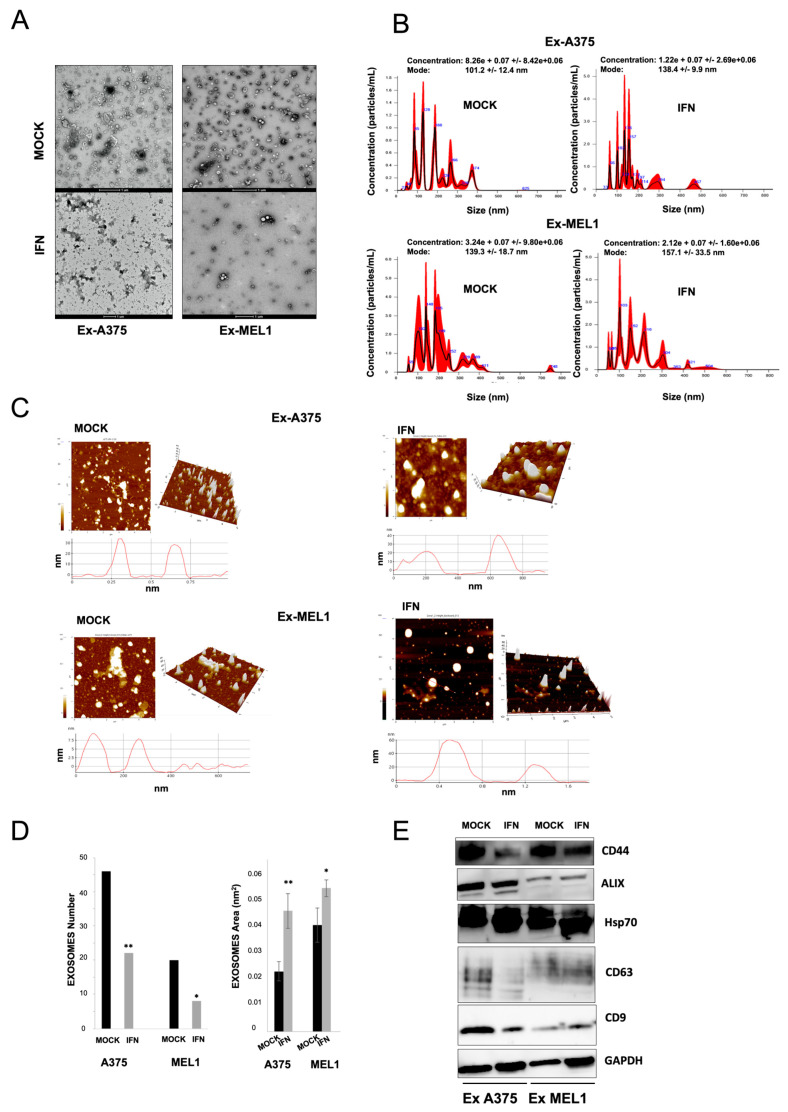
Characterization of exosomes derived from MM CSCs subpopulations under low dose IFN-α treatment. (**A**) Transmission electron microscopic images of isolated exosomes with a saucer-like shape limited by a lipid bilayer. Vesicles isolated from culture supernatant (diameter ranging from ~40 to 130 nm). (**B**) The size distribution of exosomes obtained from CSCs A375 subpopulations and CSCs MEL-1 subpopulations was analyzed by NTA. (**C**) Topography of exosomes derived from CSCs A375 subpopulations and CSCs MEL-1 subpopulations observed under atomic force microscopy (AFM). Exosomes on a mica surface revealed heterogeneity in size and shape as well as forming aggregates in both 2D (above) images and 3D profiles (below). Acquisition areas were 5 × 5 µm^2^ and 5 µm long profile lines are shown in red. (**D**) Number of exosomes counts and quantification of area from exosomes analyzed by AFM Grain Mode. (**E**) Western blot analysis of representative CD9, CD63, Alix and Hsp70 exosomes markers and the CD44 MM stem cell marker in melanospheres-derived exosomes treated under low dose IFN-α. GAPDH was used as a positive control.Statistical significance indicated * (*p* < 0.05), ** (*p* < 0.01). The full western blot figures could be found in the Supplementary Materials.

Based on their unique size and density, EVs were isolated from the culture supernatant of CSCs from A375 and MEL-1 subpopulations in the presence or absence of low dose IFN-α treatment. Exosome purification was confirmed by TEM, Western blot, NanoSight, AFM and SEM (Figure 5 and Figure S3). 

As shown in TEM images (Figure 5A), EVs obtained from CSCs—from A375 and MEL-1—showed a characteristic saucer-like ultrastructure with diameters ranging from 40 to 130 nm and crescent shaped membrane invaginations limited by a lipid bilayer, while vesicles obtained from IFN-α treated cell cultures had a minor number and diameter ranging from 30 to 90 nm. The morphology, size and organic composition of exosomes were also verified by immunogold using beads coated with an anti-CD63 antibody and scanning electron microscopy (Supplementary Figure S3). 

Exosome size distribution determined by NTA Software confirmed a decrease in particles with nanometric size in supernatants from both CSCs from A375 and MEL-1 treated with IFN-α (Figure 5B). AFM images showed a heterogeneous organization of exosomes, in terms of the wide variation in shape and size as demonstrated in both 2D images and topographic profiles, regardless of their origin. In IFN-α treated samples, we observed a low density of exosome population in comparison with exosomes released from non-treated melanospheres (Figure 5C). The number of exosomes and the quantification of area (nm^2^) and volume (nm^3^) assessed by AFM Grain Mode showed a significant increase in the counting of CSCs-mock derived exosomes in comparison to treated CSCs derived exosomes, whereas the area in exosomes derived from IFN-α treated CSCs was significantly higher and deformed than untreated CSCs for both cell lines (Figure 5D). Finally, Western blot analysis showed that these EVs from A375 and MEL-1 CSCs subpopulations were positive to known exosome classic markers including Alix, Hsp70, CD9 and CD63 (Figure 5E). Moreover, to gain additional insight into the potential role and relevance of IFN-α against CSC-melanoma-markers, CD133, p75 and CD44 markers were analyzed; however, we were able to detect only CD44 expression, which was significantly reduced in exosomes obtained from IFN-α-treated spheres (Figure 5E).

### 3.6. LC-HRMS Metabolomic Analysis of Exosomes Derived from MM CSCs Treated with IFN-α

In order to explore potential biomarkers for the diagnosis of this disease, we previously reported significant metabolomic differences in exosomes derived from melanoma CSCs from MEL-1 cell line, and also in serum-derived exosomes from melanoma patients compared to those from healthy controls [27]. Based on these findings, we were interested in checking whether some of those metabolites reported in that study were also differentially found between low dose IFN-α-treated and control CSC-derived exosome samples from A375 and MEL-1.

**Figure 6:**
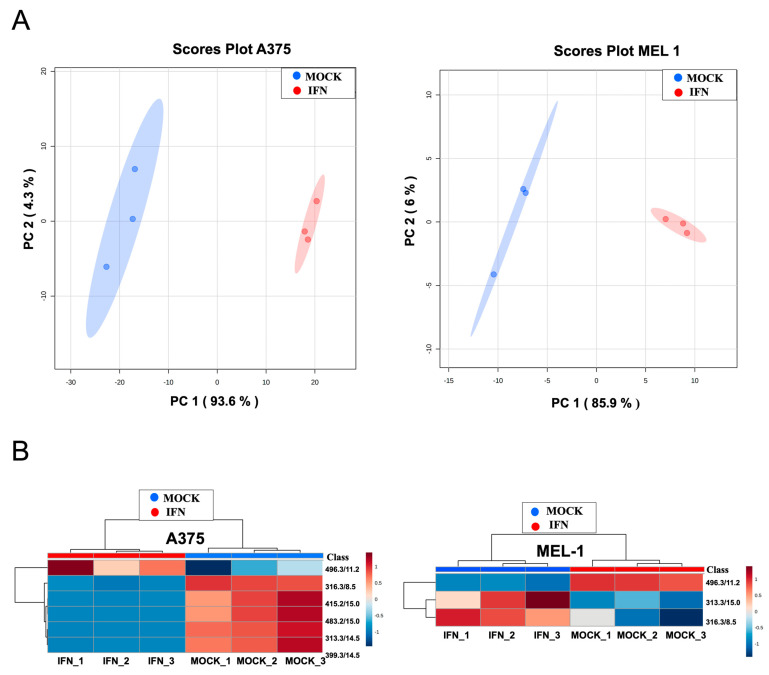
PCA score plots (**A**) and heatmaps (**B**) for all the LC-HRMS analyzed sample groups of exosomes from both MM CSCs A375 and MEL-1 subpopulations treated with IFN versus Mock control.

The PCA score plots for all the analyzed sample groups are shown in Figure 6A. In both cases, the close clustering of QC samples reflects the quality of the analytical system performance. Samples of CSC-derived exosomes treated with IFN-α were clearly separated from control samples in the PCA score plots in both cell lines (Figure 6A). In PLS-DA models, the different groups of samples were discriminated with an R2 of 0.99 and Q2 of 0.99 in A375 exosomes and an R2 of 0.99 and Q2 of 0.96 in MEL-1 exosomes. 

The corresponding heatmaps showing the differential abundance of those metabolites found as statistically different for A375 and MEL-1 exosomes are shown in Supplementary Figure S4. As previously described, we checked if some of the differential metabolites found by Palacios-Ferrer et al. [27]. Curiously, 6 and 3 of them in A375 and MEL-1 spheres, respectively, were also differentially found when comparing IFN-α-treated and mock exosome samples (Figure 6B). These signals were analyzed by PCA, obtaining a clear separation of exosome samples derived from IFN-treated and control CSCs from A375 and MEL-1 in the PCA score plots along PC1 and PC2, which describe most of the total data variability. In PLS-DA models, exosome samples were discriminated with an R2 > 0.99 and Q2 > 0.96, exceeding the threshold values accepted in metabolomic experiments (R2 > 0.7 and Q2 > 0.4) [25]. In both cell conditions, most of those metabolites were more abundant in exosome samples derived from control CSCs, compared to those from IFN-α-treated CSCs, but a unique metabolite was higher in IFN-α-treated samples in comparison to controls (Figure 6B and Figure S4). Interestingly, this metabolite with *m*/*z* 496.3381, which corresponds to 1-hexadecanoyl-sn-glycero-3-phosphocholine (PC 16:0/0:0), was previously found by our research group [27] to be overexpressed in exosomes derived from both healthy controls serum and adherent MEL-1 cells, compared to MM patients and CSC-enriched subpopulations from MEL-1 cells, respectively.

## 4. Discussion

CSCs in MM are resistant to chemo- and radiotherapy and are often involved in the recurrence of the disease. There are currently several clinical trials being conducted to test the efficacy of new selective drugs targeting this subpopulation of tumor cells. While IFN-α has been shown to potentially improve recurrence-free survival (RFS) and overall survival (OS) in patients with high-risk melanoma, its effects on OS remain questionable according to some oncologists [28]. Furthermore, adjuvant therapy with PD-1 or BRAF/MEK inhibitors has shown superior results and is being tested in combination with other options such as immunotherapy, further questioning the efficacy of IFN-α as a standalone treatment [29,30]. Despite extensive research, the exact mechanism of IFN-α action in MM remains unclear [3].

Here, we have analyzed the effects of IFN-α on aggressive CSCs established from primary MM cell lines. Our results showed inhibition in melanosphere formation and proliferation, together with a significant reduction in ALDH-positive population and in the expression of CD20, CD44 and CD133 stemness markers after IFN treatment, even at low doses (Figure 1). One of the first pieces of evidence of IFN-α effect on CSCs was shown in a rat model of ovarian cancer primary tumors, where the authors demonstrated a significant reduction of the aggressive SP after treatment [31]. Here, we have also shown such an effect for melanoma, along with the ability to inhibit cell migration, which is another important feature related to stemness (Figure 2). In agreement with our results, the anti-proliferative effects of IFN-α on triple-negative breast CSCs have also been described, associated with the presence of interferon-regulated signature genes, and improved therapeutic response and overall survival of treated patients [32]. Other studies have shown the anti-proliferative and anti-angiogenic effects of IFN-α on CSCs in hepatocellular carcinoma, and the benefit of combining IFN with other conventional therapies in different models [33].

The study conducted gene expression profiling and miRNA sequencing analysis in melanoma CSCs after treatment with low-dose IFN-α, revealing changes in the expression of genes and miRNAs related to stemness as shown in Figure 3 and Supplementary Tables S1 and S2. Up-regulated miRNAs included miR7-5p, miR141-3p, miR425, miR550a, miR3614-5p, miR4521 and miR4645, which have been shown to act on several cancer pathways as tumor suppressors. For example, miR7-5p enhances temozolomide sensitivity of drug-resistant glioblastoma cells [34], miR-425 inhibits melanoma metastasis [35] miR-3614-5p is a potential biomarker for colorectal cancer [36] and miR-4521 plays a tumor-repressive role in growth and metastasis of hepatocarcinoma cells [37]. The miRNAs miR98-5p, miR-191, miR-744-3p and let-7e-3p, known for their involvement in oncogenic mechanisms [38,39,40], were found to be down-regulated after IFN-α treatment. 

Microarray analysis revealed that IFN-α treatment led to changes in the expression of genes associated with cancer processes, including migration, apoptosis, vesicle regulation, angiogenesis, and CSC signaling pathways. These findings highlight the potential of validating gene and miRNA signatures after IFN-α treatment to identify new predictive and prognostic biomarkers for MM patients for future studies.

Moreover, we investigated the effect of IFN-α treatment on the in vivo tumourigenic capacity of melanoma CSCs (Figure 4). The results showed that IFN-α treatment at low doses significantly decreased the ability of CSCs to form tumors. The immunoanalysis of tumors revealed a significant increase in the expression of SAMD9L according to data obtained from previous microarrays analysis. SAMD9L is a protein with antiproliferative function and a tumor suppressor role in various types of cancer, recently identified in genetic signatures of pancreas and melanoma tumors [41]. While it regulates cell proliferation in hematopoietic tissue by facilitating the degradation of cytokine receptors, its role in stemness processes and cancer needs further analysis. These findings suggest that IFN-α treatment may be a potential therapeutic strategy for melanoma, and further research on the role of SAMD9L in melanoma and other cancers is warranted.

Extracellular vesicles (EVs) have been shown to play a role in cancer progression and the metastatic process by altering the microenvironment of distant sites, promoting angiogenesis and tumor cell migration [42]. EVs can be isolated by non-invasive liquid biopsy and used as a source of prognostic and predictive biomarkers. In this study, we found that IFN-α treatment decreased EV secretion via exosomes in melanoma CSC subpopulations. Exosomes from melanoma CSCs express the CD44 CSC-related marker, and this expression was reduced after treatment (Figure 5). The microarrays analysis we conducted also revealed a modulation in mRNA expression of several members of the VAMP family after IFN-α treatment, suggesting their potential involvement in exosome dysregulation since VAMP1 and VAMP8 are essential components of the exocytic machinery and regulate various secretory processes in different systems [43]. Few recent studies have demonstrated the effect of IFN-gamma on vesicular trafficking from neural CSCs by inducing the generation of altered exosomes [44]. Further research is necessary to identify the underlying mechanisms responsible for the modulation of CSC-derived exosomes and their potential relationship with the efficacy of IFN-α treatment.

Our previous study, focused on searching potential biomarkers for the diagnosis of melanoma, reported significant metabolomic differences in exosomes derived from MM CSCs compared to those from differentiated tumor cells in A375 and MEL-1 cell line [27]. We show in this present work the differential abundance of those metabolites in exosomes from IFN-treated CSC-A375 and CSC-MEL1 (Figure 6). Interestingly, the metabolite with *m*/*z* 496.3381, which corresponds to 1-hexadecanoyl-sn-glycero-3-phosphocholine (PC 16:0/0:0), was previously found by Palacios-Ferrer and cols. [27] to be expressed in exosomes derived from both healthy control serum and adherent MEL-1 cells, compared to MM patients’ serum and CSC-enriched subpopulations from MEL-1 cells, respectively. Curiously, PC 16:0/0:0 was higher in IFN-α -treated samples in comparison to controls. The over-expression of this metabolite can be in accordance with previous studies demonstrating its relationship with lower risks of breast, prostate and colorectal cancer [45]. In fact, it has been suggested that the rapid extracellular hydrolysis of phospholipids like PC 16:0/0:0 by metastatic tumor cells and the subsequent cellular uptake of the resulting free fatty acids (FFA) seems to be a necessary prerequisite for metastatic potential of epithelial tumor cells, probably for generating pro-metastatic lipid second messengers [27,46]. 

## 5. Conclusions

The results of this present study demonstrate that IFN-α has a significant effect on MM CSC-enriched subpopulations, as evidenced by various stem cell characterizations performed, including a reduction in tumor formation. Furthermore, IFN-α treatment modulates the metabolic and proteomic composition of MM CSC-derived exosomes, suggesting the potential for identifying biomarkers at this level. However, the consequences of exosome modulation on tumor communication by IFN-α need to be evaluated. 

Since the majority of the anti-CSC effects observed in this study were achieved with low doses of IFN-α, it suggests the benefits of continuing to explore IFN-α-based therapy in MM patients, particularly in combination with novel therapeutic approaches such as immunotherapies or targeted therapies.

## Data Availability

All data needed to replicate our analyses are available upon request for the corresponding authors.

## Supplementary Materials

The following supporting information can be downloaded at: https://www.mdpi.com/article/10.3390/cancers15143666/s1, Figure S1: Effects of IFN-α on CSCs cell cycle and apoptosis; Figure S2: Array Analysis and Western Blot of genes in both cell lines; Figure S3: Characterization of exosomes derived from melanospheres cultures under IFN-α conditions; Figure S4: Heatmaps representing the differential abundance of metabolites shown for CSCs A375 (up) and MEL-1 (down) derived exosomes; Table S1: List of the selected genes whose expression has changed after 24 h of IFN treatment in A375 CSCs; Table S2: List of the selected genes whose expression has changed after 24 h of IFN treatment in MEL-1 CSCs. Supplementary Figures: The full western blot figures.
