# Supplementary material for: Interferon-Alpha Decreases Cancer Stem Cell Properties and Modulates Exosomes in Malignant Melanoma
Source: Cancers (Basel). 2023 Jul 18;15(14):3666. doi: 10.3390/cancers15143666 (PMC10377490; doi:10.3390/cancers15143666)
Supplement: Supplementary file 1 [file cancers-15-03666-s001.zip › cancers-2453986-supplementary.pdf]

Supplemental Material

Interferon-Alpha Decreases Cancer Stem Cell Properties and Modulates Exosomes in Malignant Melanoma

Supplemental Material

Materials and Methods

Cell cycle analysis

Cell cycle analysis was performed using the propidium iodide assay. After 3 days in different culture IFN-α conditions, melanospheres were harvested, washed twice with PBS, and fixed in 70% (vol/vol) cold ethanol for up to 1 week. Next, the cells were centrifuged, and the pellet was washed once with phosphate-buffered saline and resuspended in 250μL of propidium iodide solution (100μL/mL RNAse, 40μL/mL propidium iodide in phosphate-buffered saline) for 30 minutes in the dark at 37°C. All samples were analyzed in a FACS Canto II cytometer (BD Biosciences).

Apoptosis

After 3 days in different IFN-α dose, MM CSCs were washed and mechanically disrupted with a pipette and by syringing three to five times through a sterile 21-gauge needle. After, spheres were analyzed using an Annexin V-fluorescein isothiocyanate detection kit (eBioscience Inc.). All samples were analyzed in a FACS Canto II cytometer (BD Biosciences).

Quantitative real time-PCR (qRT-PCR)

To confirm reproducibility of miRNA expression profiles obtained by miRNAseq, qRT-PCR validation experiments were carried out. Technical and biological replicates of RNA were prepared for untreated cells and after IFN-α stimulation for 30 h. cDNA was synthesized by reverse transcription of total RNA using the Reverse Transcription System

(Promega) for mRNA, and miRCURY LNA TM Synthesis kit II (Exiqon) for miRNAs. qRT-PCR assay was done using SYBR Green PCR Master Mix (Promega) and miRCURY LNA TM EXILENT SYBR Green (Exiqon) for miRNAs. Each experiment was performed in duplicate and reactions were performed in triplicate. The comparative threshold cycle (Ct) method was used to calculate the amplification factor as specified by the manufacturer ABI 7500. QRT-PCR consisted of 45 cycles of 95 °C for 10 sec, 60 °C for 40 sec, and 72 °C for 1 sec, after an initial denaturation step (95 °C for 10 min). Expression levels were normalized to SNORD44 and U6 as the internal controls and quantified by the comparative Ct (ΔΔCt) method. Primer sequences are listed in Annex I (Supplementary data Table S2 in Annex II).

Transmission and Scanning Electron Microscopy

Transmission Electron Microscopy (TEM) and Scanning Electron Microscopy (SEM) analyses were performed at the Scientific Instrumental Centre (CIC-University of Granada). For TEM and SEM samples were negatively stained with uranyl acetate as follows: a 30 µL drop of the exosome sample was placed on a carbon-coated 300 mesh grid and allowed to adsorb at room temperature for 5 min. The grids were then washed in drops of ultrapure water for 1 min. Adsorbed exosomes were negatively stained by placing the grids on a drop of 1% uranyl acetate in aqueous suspension for 1 min. The excess of fluid was drained with filter paper slightly, and then sample grids were dried at room temperature for 6 min. The preparations were examined with a LIBRA 120 PLUS transmission electron microscope (Carl Zeiss SMT, Oberkochen, Germany) at an acceleration voltage of 120 kV and the HITACHI, S-510 scanning electron microscope. Then, samples were determined with the Edwin-Róntec microanalysis system (Palacios-Ferrer et al., 2021). In addition, pellets obtained from CSCs cultures were immersed in 4% paraformaldehyde, 0.1 M PBS for 4 hours at 4ºC and washed in sucrose in 0.1 M PBS

overnight. The fractions were incubated by increase alcohol concentrations and were cut in semithin sections at 0.5 μm with tissue processor (TP1020, Leica, Germany).

Atomic Force Microscopy

Atomic Force Microscopy (AFM) analyses were performed at the Scientific Instrumental Centre (CIC-University of Granada). For AFM purified exosomes were diluted 1:10 in deionized water. A 10μL drop of exosome suspension was adsorbed to freshly cleaved mica sheets at room temperature for 10 min and rinsed with deionized water to remove salt precipitates. The sheets were then completely dried under a gently stream of argon gas (Ar). The preparations were examined with a NX20 Atomic Force Microscope (Park Systems, Suwon, South Korea) and images were visualized and processed using the Park Systems XEI software. Measurements were carried out with ACTA cantilevers (40 N·m-1) and in Non Contact Mode (Palacios-Ferrer et al., 2021).

To collect supernatants, we used the same volumen in each purification procedure (15 mL) and we adjusted the initial number of cells taking into account the different degree of growth in the presence or absence of IFN-α, in order to have the same total number of cells for both conditions (8×10^6 cells) at the time of collection of the supernatants.

Inmunogold Labeling by Transmission Electron Microscopy

Inmunogold labeling of exosomes was performed at the Andalusian Centre for Nanomedicine (Bionand, Spain). Exosomes suspensions were put on copper grid with Formvar-Carbon and incubate 15 min at RT. Dried slightly and was passed to a drop of 15 μl of 2% paraformaldehyde in 0.1 M PBS and incubate 10 min. The samples were transferred to a drop of 15 μL of 2% BSA in 0.1 M PBS, plus the primary antibody Anti-Human CD63 Clone H5C6 (RUO) (Becton Dickinson) diluted 1/500 and incubated 1.5 hour at room temperature and in a humid chamber (Palacios-Ferrer et al., 2021). After several PBS washed, the grid was incubated with secondary antibody Anti-Mouse IgG

(Whole molecule)-gold antibody 10 nm (Sigma Aldrich) and incubated 1 hour at room temperature and in a humid chamber. The samples were made with a negative stain by passing a drop of 15 µL of 1% uranyl acetate in Milli-Q water for 15 seconds and the preparations were examined with a LIBRA 120 PLUS transmission electron microscope (Carl Zeiss SMT, Oberkochen, Germany).

LC-HRMS analysis of exosomes

The HPLC-QTOF-MS total ion chromatograms (TIC) showed excellent reproducibility with regards to retention time and signal intensity, indicating a low analytical drift across the whole set of samples (data not shown). A positive ionization data matrix of 4300 mass signals was obtained as an outcome of the peak picking and alignment procedures, but only 676 peaks representing monoisotopic ions were considered and subjected to the chemometric analysis. A first filtering process was performed in order to discard those signals coming from mobile phase solvents.

Next, multivariate analyses such as PCA and PLS-DA were carried out in order to assess the quality of the analytical system performance and discriminate those variables that are responsible for variation between the comparison groups.

After that, a T-test (p-value < 0.05) filtering was performed for comparison on the 2 groups of samples (IFN-α-treated and mock treated CSC-derived exosomes) for both MM cell lines. As an outcome, 73 and 76 differential metabolites were found in A375 and MEL-1 exosomes, respectively.

SUPPLEMENTARY FIGURE CAPTIONS

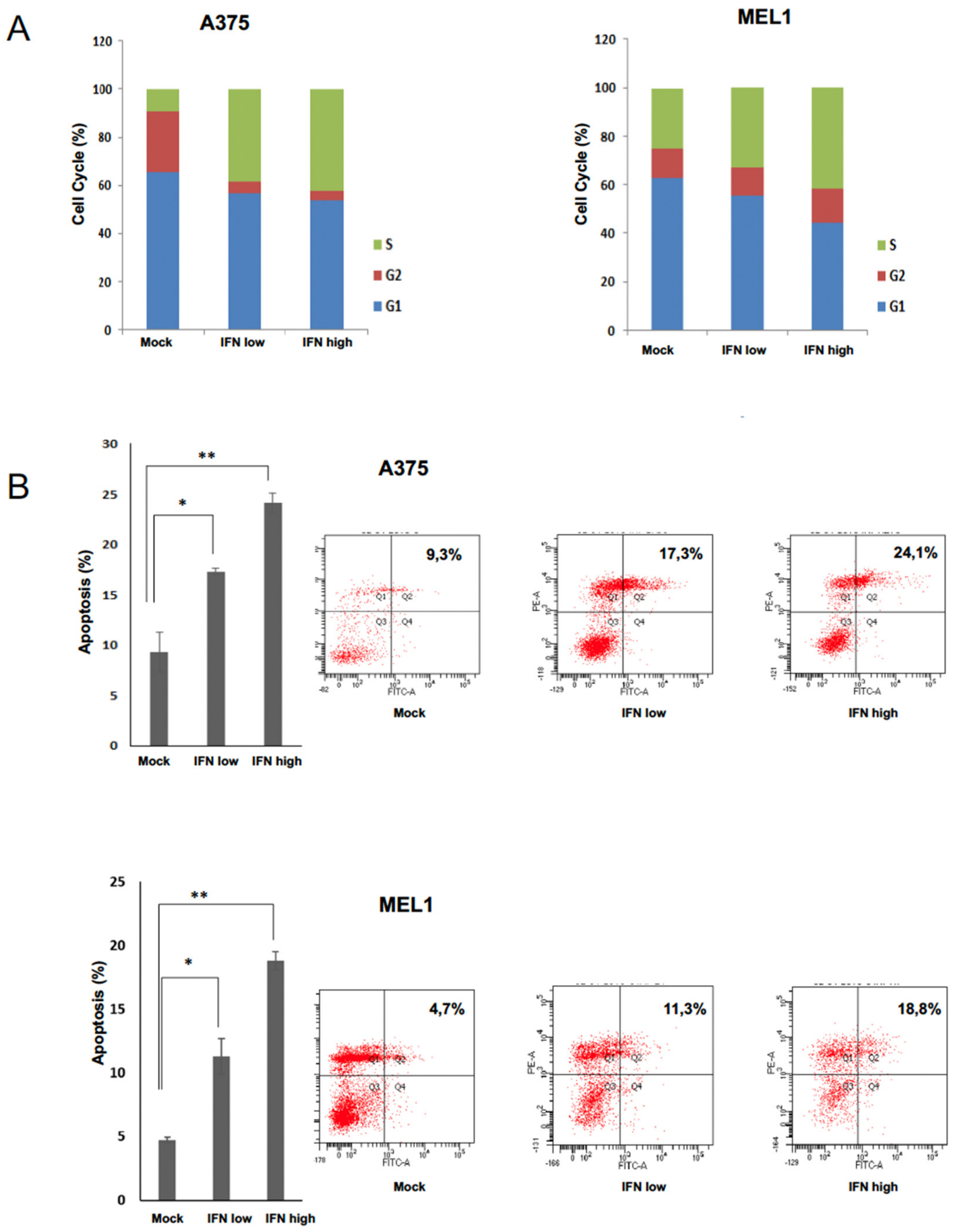

Figure S1

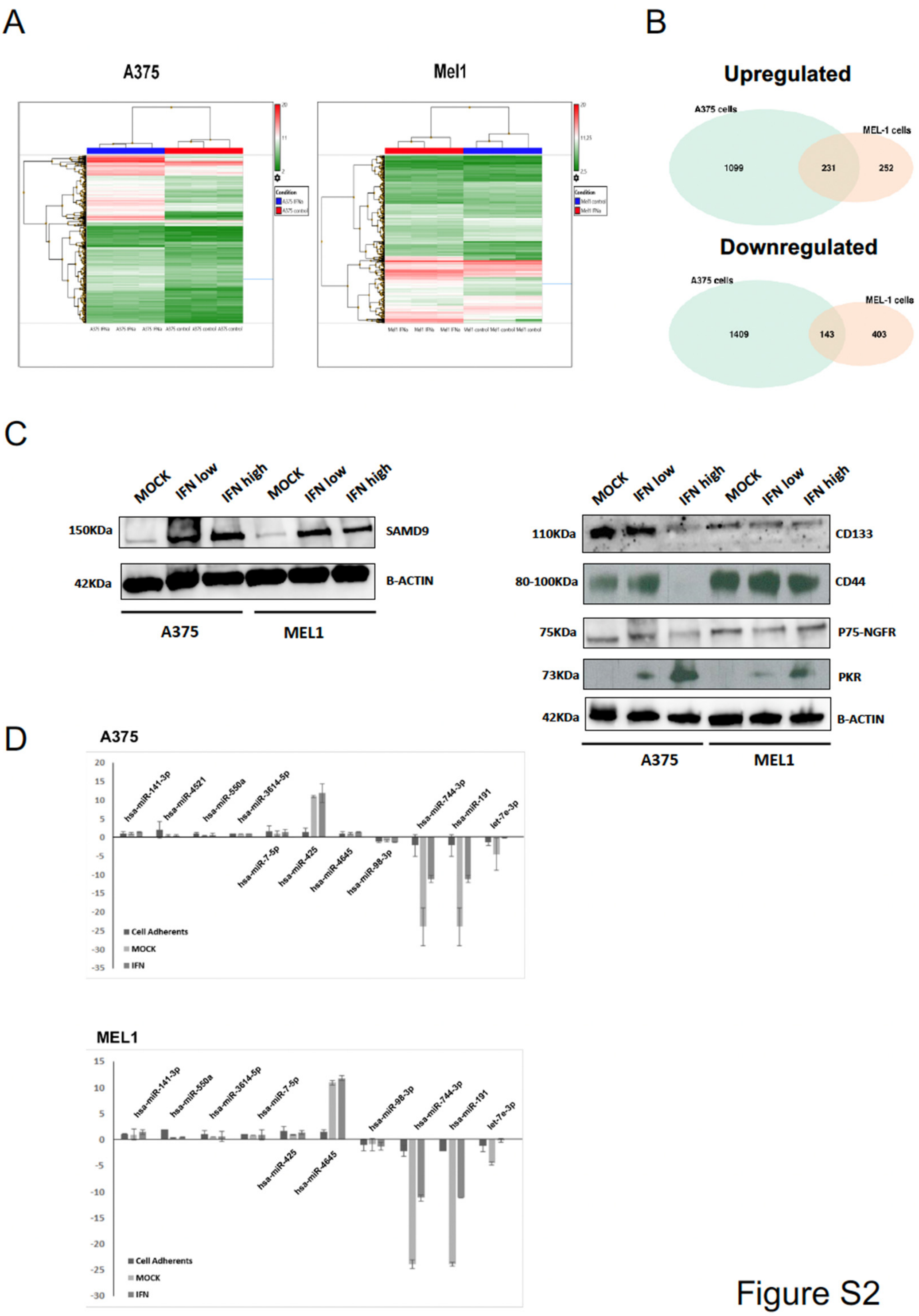
A
A375
Mel1
B
Upregulated
A375 cells
MEL-1 cells
1099
231
252
Downregulated
A375 cells
MEL-1 cells
1409
143
403
C
MOCK
IFN low
IFN high
MOCK
IFN low
IFN high
150KDa
SAMD9
42KDa
B-ACTIN
A375
MEL1
110KDa
CD133
80-100KDa
CD44
75KDa
P75-NGFR
73KDa
PKR
42KDa
B-ACTIN
A375
MEL1
D
A375
hsa-miR-141-3p
hsa-miR-4521
hsa-miR-550a
hsa-miR-3614-5p
hsa-miR-7-5p
hsa-miR-425
hsa-miR-4645
hsa-miR-98-3p
hsa-miR-744-3p
hsa-miR-191
let-7e-3p
Cell Adherents
MOCK
IFN
MEL1
hsa-miR-141-3p
hsa-miR-550a
hsa-miR-3614-5p
hsa-miR-7-5p
hsa-miR-425
hsa-miR-4645
hsa-miR-98-3p
hsa-miR-744-3p
hsa-miR-191
let-7e-3p
Cell Adherents
MOCK
IFN

Figure S2

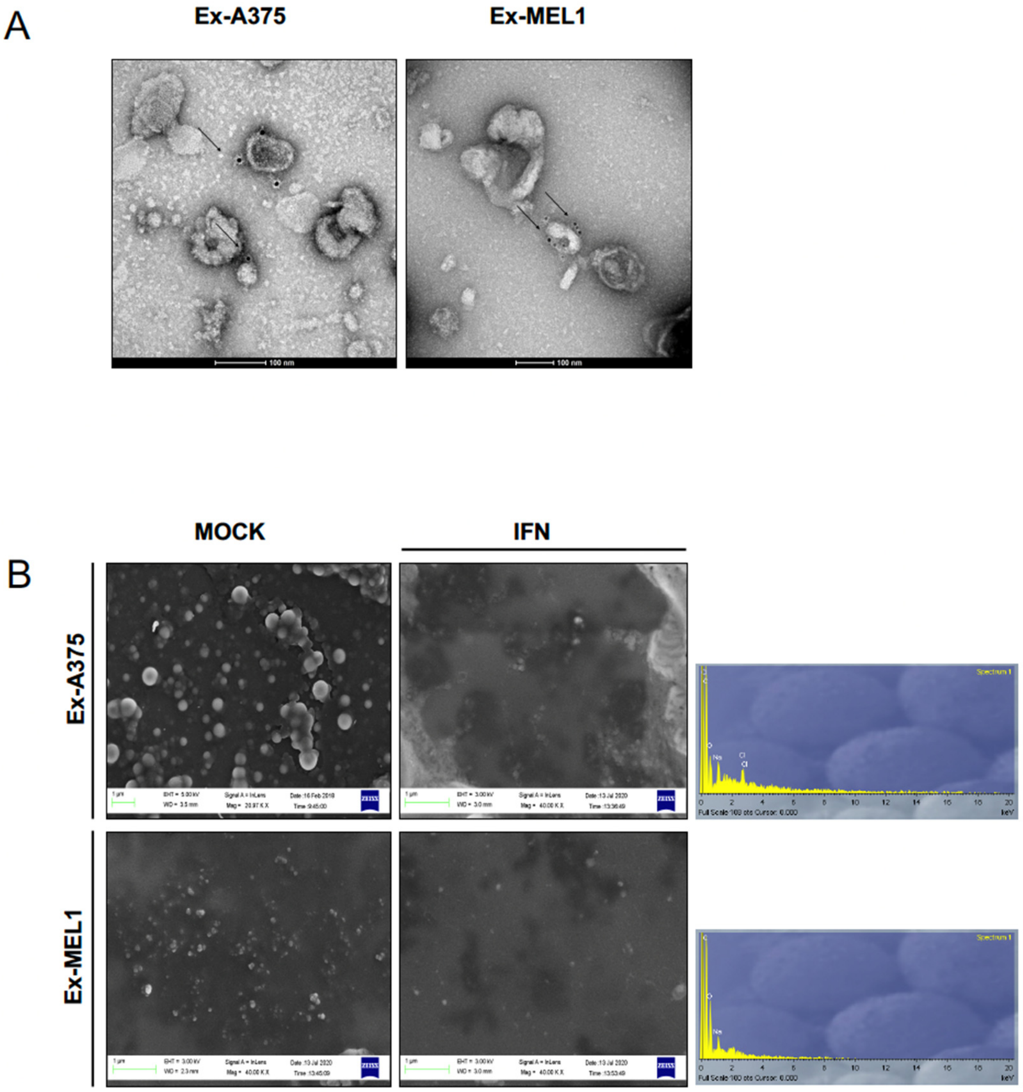
A
Ex-A375
Ex-MEL1
100 nm
100 nm
B
MOCK
IFN
Ex-A375
Ex-MEL1
Spectrum 1
O
Na
Cl
Cl
Full Scale 900 cts Cursor: 0.000
keV

Figure S3

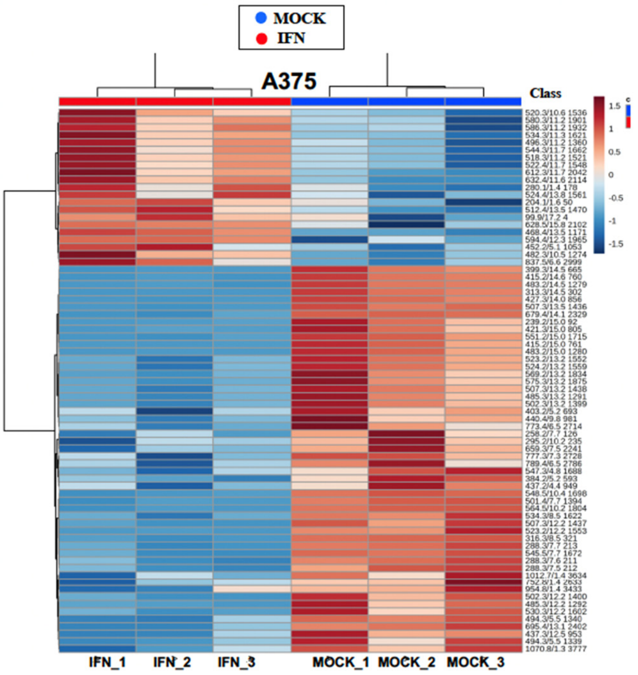
MOCK
IFN
A375
Class
IFN_1
IFN_2
IFN_3
MOCK_1
MOCK_2
MOCK_3

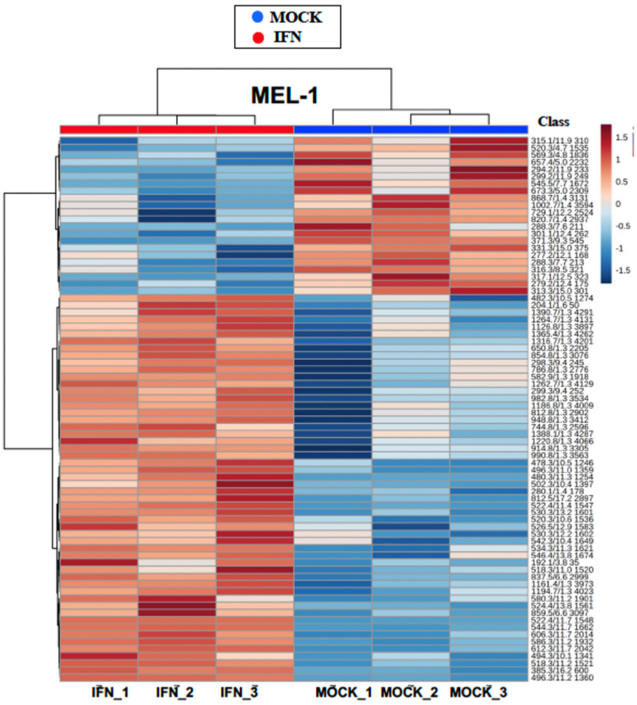
MOCK
IFN
MEL-1
Class
IFN_1
IFN_2
IFN_3
MOCK_1
MOCK_2
MOCK_3

Figure S4

Figure S1. Effects of IFN-α on CSCs cell cycle and apoptosis. (A) Cell cycle analysis in CSCs enriched subpopulations. Flow cytometry analysis of cell cycle distribution of A375 and MEL1 cells; (B) Apoptosis assay in CSCs enriched subpopulations. Cell viability and apoptosis analysis of cells A375 and MEL1 after treatment with IFN-α; Scatter representation of PI/Annexin V analysis. Statistical significance indicated *($p < 0.05$), **($p < 0.01$), *** ($p < 0.001$) and # ($p < 0.0001$).

Figure S2. Array Analysis and Western Blot of genes in both cell lines. (A) Expression profile heatmap of the fold change genes up and downregulated after 24h of IFN treatment in A374 and MEL-1 CSCs; (B) Number of common up- and downregulated genes (Fold Change > 2 and FDR < 0.05) between A375 and MEL-1 cells after 24h of IFN-α treatment; (C) Western blot analysis of representative SAMD9, CD133, CD44, p75 and PKR markers in melanospheres-derived exosomes treated under IFN-α conditions. B-Actin was used as a positive control.; (D) Expression values of selected miRNAs in A-375 and MEL-1 CSCs.

Figure S3. Characterization of exosomes derived from melanospheres cultures under IFN-α conditions. (A) Inmunogold with Anti-CD63 marker of exosomes derived from spheres secondary cultures in both cell lines. Black arrows point to exosomes; (B) Scanning electron microscopy images of CSCs derived-exosomes aggregated from both A375 (up) and MEL-1 spheres supernatants (down). The micro-analysis determined the proportion of carbon and discriminated the sales.

Figure S4. Heatmaps representing the differential abundance of metabolites shown for CSCs A375 (up) and MEL-1 (down) derived exosomes.

SUPPLEMENTARY TABLES CAPTIONS

Table S1. List of the selected genes whose expression has changed after 24h of IFN treatment in A375 CSCs

ID	A375 IFN-α Avg (log2)	A375 MOCK Avg (log2)	Fold Change	P-val	FDR P-val	Gene Symbol	Description
TC0700011797.hg.1	15,03	5,99	529,84	7,83E-14	1,62E-11	SAMD9L	sterile alpha motif domain containing 9-like
TC1400008056.hg.1	12,71	4,09	392,9	3,66E-14	9,80E-12	IFI27	interferon, alpha-inducible protein 27
TC0200015242.hg.1	18,26	12,31	61,63	2,59E-13	3,71E-11	STAT1	signal transducer and activator of transcription 1
TC1200010908.hg.1	12,43	7,33	34,33	1,90E-13	2,91E-11	STAT2	signal transducer and activator of transcription 2
TC0900006559.hg.1	8,73	3,8	30,47	1,28E-13	2,39E-11	CD274	CD274 molecule
TC0300013146.hg.1	6,86	3,24	12,29	1,54E-09	3,33E-08	TNFSF10	tumor necrosis factor (ligand) superfamily, member 10
TC0200010447.hg.1	11,53	7,93	12,15	1,47E-13	2,62E-11	CASP8	caspase 8, apoptosis-related cysteine peptidase
TC0600013231.hg.1	12,33	9,79	5,85	7,48E-09	1,19E-07	SGK1	serum/glucocorticoid regulated kinase 1
TC1200009734.hg.1	8,46	6,09	5,14	1,34E-06	9,29E-06	VAMP1	vesicle associated membrane protein 1
TC0200010445.hg.1	6,74	4,66	4,23	6,82E-07	5,29E-06	CASP10	caspase 10
TC0900008202.hg.1	9,63	7,73	3,74	4,89E-06	2,81E-05	TGFBR1	transforming growth factor, beta receptor 1
TC1000008954.hg.1	9,1	7,26	3,56	1,16E-05	5,94E-05	CASP7	caspase 7
TC1100013221.hg.1	5,48	3,93	2,92	0,0143	0,0284	CASP1	caspase 1
TC1100012165.hg.1	11,6	10,55	2,07	0,0003	0,001	CASP4	caspase 4
TC1700011749.hg.1	11,51	10,61	1,87	0,0007	0,0022	GRB2	growth factor receptor bound protein 2

TC0100011533.hg.1	5	7,74	-6,68	5,61E-09	9,43E-08	ATF3	activating transcription factor 3
TC0100014349.hg.1	6,97	11,16	-18,25	1,74E-13	2,85E-11	JUN	jun proto-oncogene

Table S2. List of the selected genes whose expression has changed after 24h of IFN treatment in MEL-1 CSCs.

ID	Mel1 IFN-α Avg (log2)	Mel1 MOCK Avg (log2)	Fold Change	P-val	FDR P-val	Gene Symbol	Description
TC0700011797.hg.1	14,8	6,55	305,22	7,31E-13	1,12E-09	SAMD9L	sterile alpha motif domain containing 9-like
TC1400008056.hg.1	13,08	6,6	89,31	2,46E-11	1,89E-08	IFI27	interferon, alpha-inducible protein 27
TC0300013146.hg.1	13,62	8,18	43,66	2,11E-13	4,83E-10	TNFSF10	tumor necrosis factor (ligand) superfamily, member 10
TC0900006559.hg.1	8,86	5,08	13,69	1,14E-11	1,06E-08	CD274	CD274 molecule
TC0200015242.hg.1	18,71	15,78	7,62	5,38E-08	1,16E-05	STAT1	signal transducer and activator of transcription 1
TC1200010908.hg.1	11,59	8,7	7,4	2,25E-09	8,19E-07	STAT2	signal transducer and activator of transcription 2
TC1100013221.hg.1	5,77	3,35	5,34	4,24E-05	0,0027	CASP1	caspase 1
TC1100012165.hg.1	11,65	9,63	4,07	2,09E-08	5,00E-06	CASP4	caspase 4
TC1000008954.hg.1	10,21	8,41	3,48	5,15E-05	0,0031	CASP7	caspase 7
TC0100011533.hg.1	6,68	5,21	2,77	1,15E-05	0,001	ATF3	activating transcription factor 3
TC0200010445.hg.1	5,64	4,21	2,68	0,0002	0,0087	CASP10	caspase 10
TC0200010447.hg.1	12,27	11,12	2,22	0,0001	0,0057	CASP8	caspase 8, apoptosis-related cysteine peptidase
TC0600013231.hg.1	14,24	13,11	2,2	0,0002	0,0085	SGK1	serum/glucocorticoid regulated kinase 1

TC0100014349.hg.1	6,02	4,89	2,18	0,001	0,0259	JUN	jun proto-oncogene
TC1700011749.hg.1	11,17	11,32	-1,11	0,6776	0,8887	GRB2	growth factor receptor bound protein 2
TC1200009734.hg.1	6,85	8,25	-2,63	0,0013	0,032	VAMP1	vesicle associated membrane protein 1
TC0900008202.hg.1	8,43	10,19	-3,38	5,08E-05	0,0031	TGFBR1	transforming growth factor, beta receptor 1

References:

Palacios-Ferrer, J. L., García-Ortega, M. B., Gallardo-Gómez, M., García, M. Á., Díaz, C., Boulaiz, H., Valdivia, J., Jurado, J. M., Almazan-Fernandez, F. M., Arias-Santiago, S., Amezcua, V., Peinado, H., Vicente, F., Pérez del Palacio, J., & Marchal, J. A. (2021). Metabolomic profile of cancer stem cell-derived exosomes from patients with malignant melanoma. *Molecular Oncology*, *15*(2). https://doi.org/10.1002/1878-0261.12823
