# Supplementary figures and images for: Interferon-Alpha Decreases Cancer Stem Cell Properties and Modulates Exosomes in Malignant Melanoma
Source: Cancers (Basel). 2023 Jul 18;15(14):3666. doi: 10.3390/cancers15143666 (PMC10377490; doi:10.3390/cancers15143666)

SAMD9L

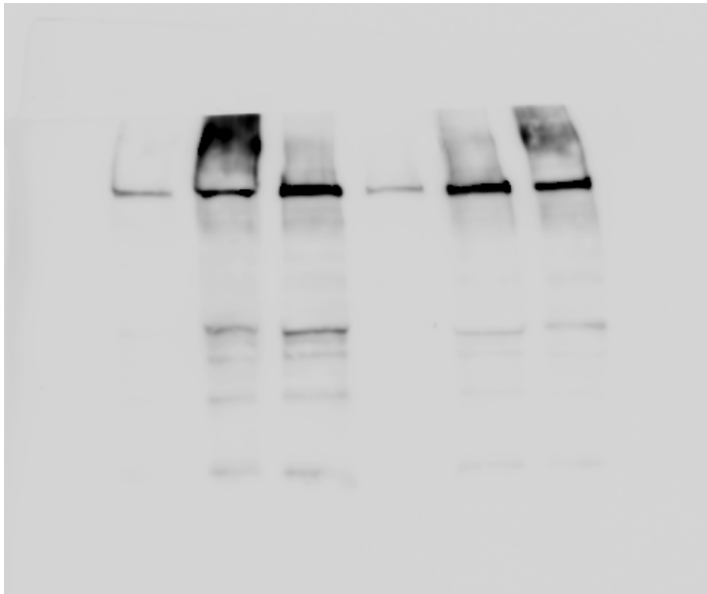

CD133

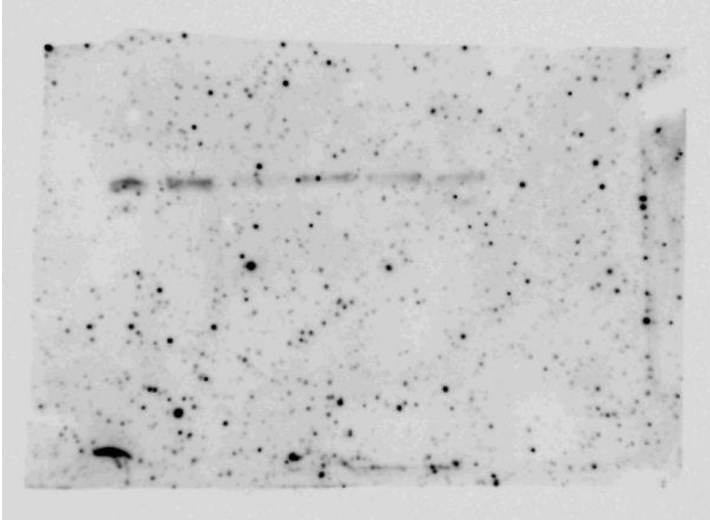

**Figure S2**

PKR

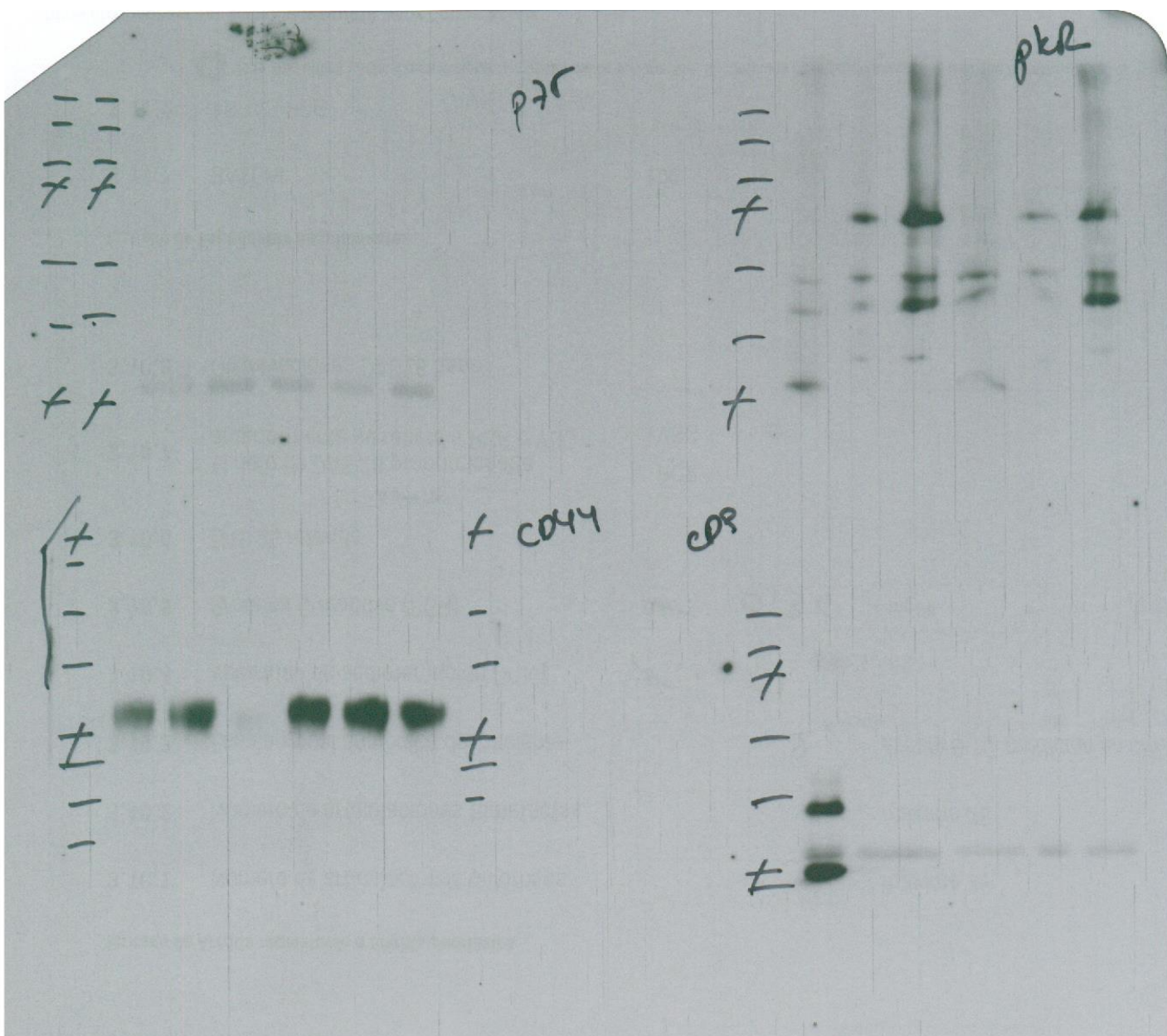

CD44

Figure S2

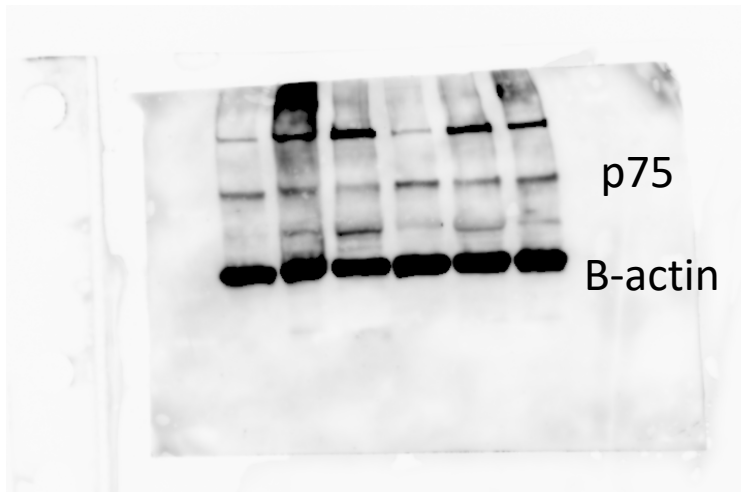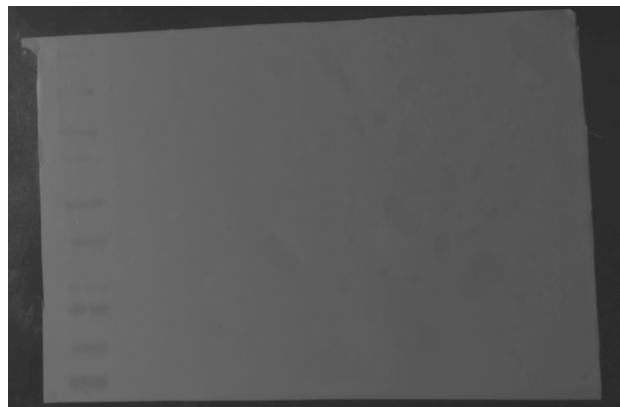

standar  
weight

**Figure S2**

Alix

GADPH

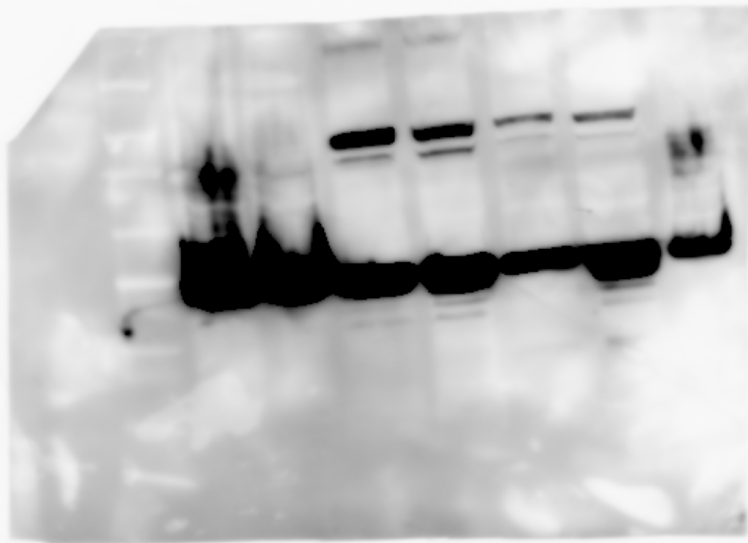

CD63

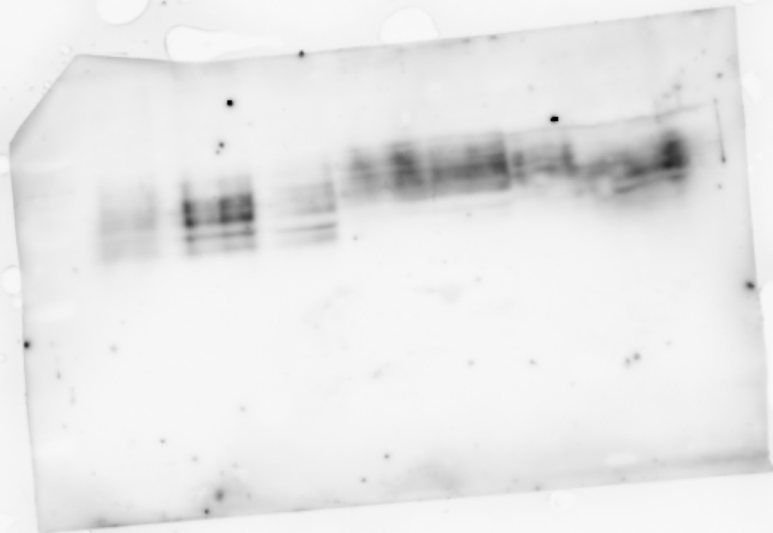

**Figure 5**

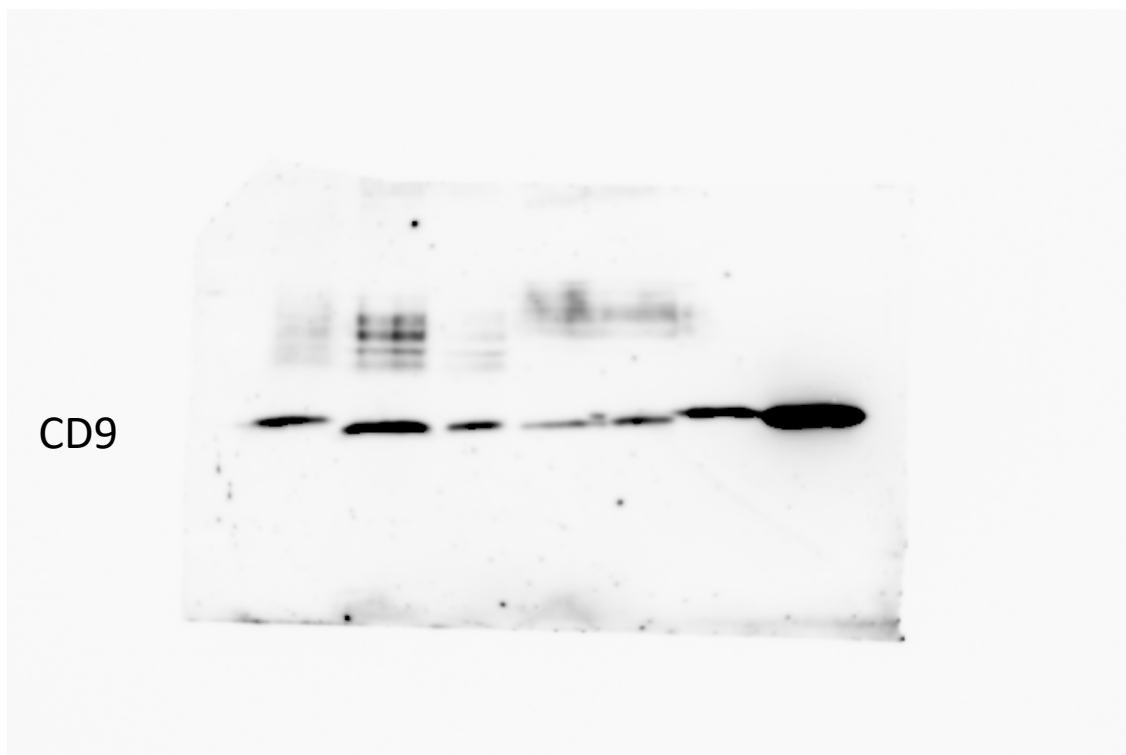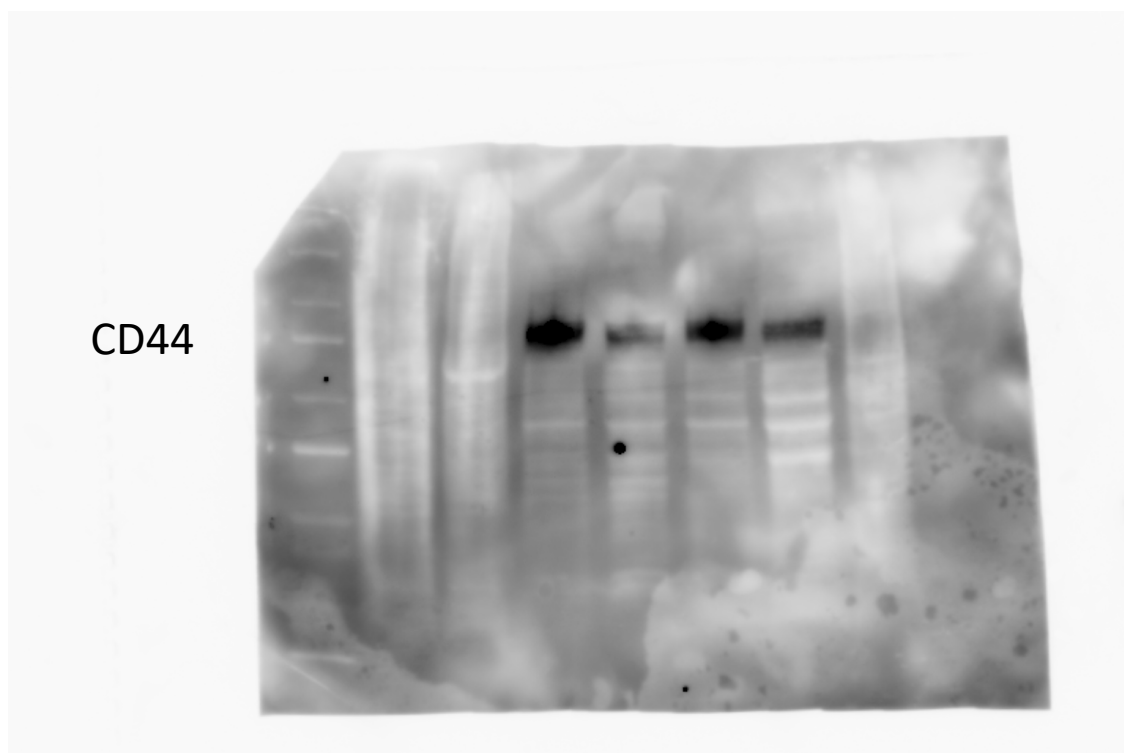

**Figure 5**

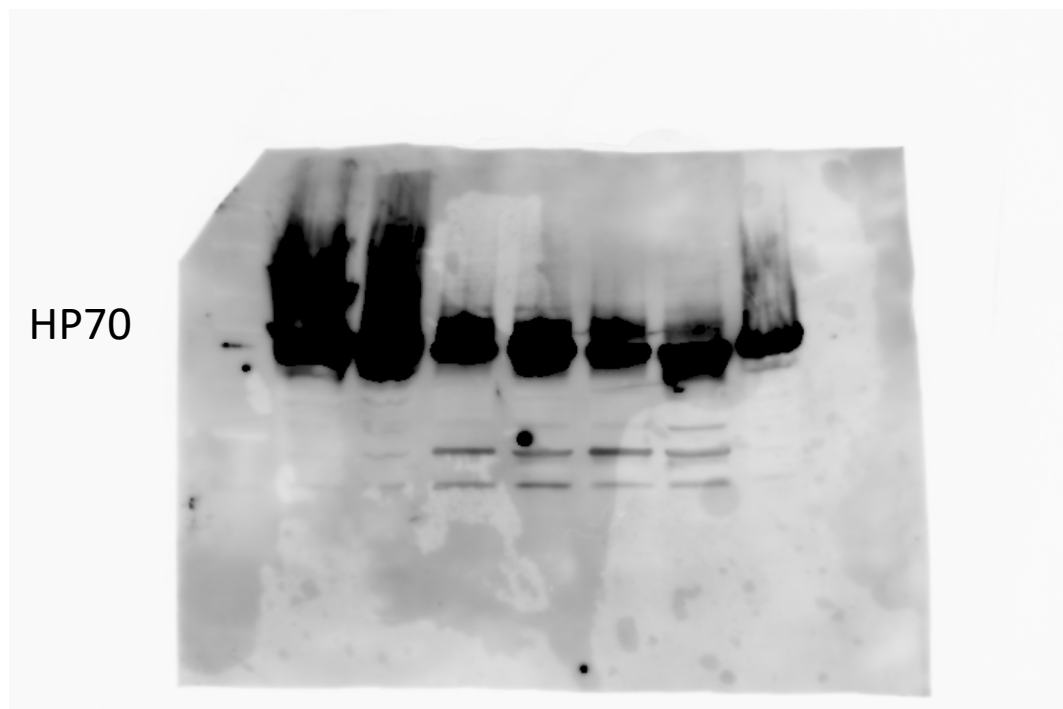

**Figure 5**

Supplement: Supplementary file 1 [file cancers-15-03666-s001.zip › cancers-2453986-original-images.pdf]
